# *Bacillus* spp. in Fish Farming: Enhancing Fish Performance, Health and Farming Practices

**DOI:** 10.1155/anu/3323307

**Published:** 2026-05-15

**Authors:** Yaoying Lu, Natasha B. Bambridge, Xiaojing Chen, Yunjiang Feng

**Affiliations:** ^1^ Institute for Biomedicine and Glycomics, Griffith University, Parklands Drive, Gold Coast, 4222, Queensland, Australia, griffith.edu.au; ^2^ Bioproton Pty Ltd., 55 Dulacca Street, Brisbane, 4110, Queensland, Australia; ^3^ School of Environment and Science, Griffith University, 170 Kessels Road, Brisbane, 4111, Queensland, Australia, griffith.edu.au

**Keywords:** aquaculture, *Bacillus* spp, feed supplements, fish farming, probiotics

## Abstract

Aquaculture has become a crucial component of global food and nutrition security, offering a sustainable alternative to meet the growing demand for seafood while alleviating pressure on wild fish populations. Among various strategies to enhance aquaculture productivity, probiotics have gained considerable attention for their ability to improve fish growth performance, overall health and environmental farming conditions. This review explores the potential of *Bacillus* spp. as probiotics in fish farming, with particular emphasis on *B. subtilis*, *B. licheniformis*, *B. amyloliquefaciens*, *B. velezensis*, *B. pumilus*, *B. coagulans* and *B. megaterium*. Evidence indicates that *Bacillus* spp. can enhance fish growth performance by improving feed efficiency, promoting gut health and innate immune responses and contributing to improved water quality through enhanced nutrient cycling and reduced environmental pollutants. Among these species, *B. subtilis* emerges as the most extensively studied and applied, likely due to its robustness, abundance, safety and multifunctional probiotic properties. In contrast, other *Bacillus* spp. remain relatively underexplored, despite their potential to provide complementary or strain‐specific benefits. This review also highlights key knowledge gaps, including the limited understanding of probiotic mechanisms at the metabolomic level. Future research integrating multi‐omics approaches and evaluating both single‐ and multi‐strain *Bacillus* probiotics will be essential for optimising probiotic design and advancing sustainable aquaculture practices.

## 1. Introduction

Aquaculture is increasingly important for addressing global food and nutrition security. As demand for seafood rises, aquaculture provides a sustainable way to meet this need without depleting wild fish populations, and to protect ecosystems and biodiversity. Aquatic animal products are also key sources of protein and essential nutrients, particularly in regions where access to other animal proteins is limited. In 2022, aquaculture production reached record levels, contributing significantly to global protein supplies, especially in Asia and Africa [1]. Additionally, the most traded aquatic animal products in 2022 were finfish (65%), crustaceans (23%), molluscs and other aquatic invertebrates (11%), indicating more effort can be given to fish farming to meet the growing demand and to ensure its sustainability [1].

Fish farming is unique in comparison to terrestrial farming: the fish are immersed in their farming environment creating a more intimate relationship between the fish and the water [2, 3]. Water is an ecosystem filled with diverse microorganisms varying from eukaryotes, commensal bacteria and pathogens which influence the health of the fish [3]. While harmful microorganisms are present in aquaculture, there has been an exploration into the application of beneficial microorganisms, or probiotics, into the farming environment to improve the fish’s health directly or indirectly [2–4].

Fish are a diverse animal group with different tolerances to water temperature, salinity and oxygen levels in the water, all of which influence the selection of probiotics [2]. When choosing probiotics for fish farming, several critical factors must be considered. First, suitable probiotics must be safe, posing no harm to the fish or to any potential consumers [4, 5]. They should also be ingestible by fish, capable of surviving passage through the digestive system, able to withstand the harsh conditions of the stomach, bile and pancreas, and ideally colonise within the host to prolong their beneficial effects [2, 5]. Second, it is imperative that the probiotics are effective in enhancing fish growth. Probiotics promote growth through various mechanisms, including directly stimulating growth, improving overall health and preventing diseases [2–7]. Third, probiotics contribute to improved water quality. Numerous studies have shown that bacterial‐based probiotics can reduce organic matter and nitrogenous compounds, such as ammonia and nitrite, as well as phosphorus and hydrogen sulphide levels in water [2, 4–7]. Finally, probiotics also play a significant role in pathogen reduction. By decreasing the number of harmful microorganisms in the water, probiotics lower the risk of infections for fish [3–6]. The reduction of pathogens is not limited to water applications; probiotics administered to the fish also reduce harmful pathogens in fish meat and gut, while increase the immune response to potential pathogens, thus decreasing the chance of disease [2–5, 7].

Probiotics are defined as ‘live microorganisms which when administered in adequate amounts confer a health benefit on the host’ [8, 9]. While probiotics are primarily bacteria, they also include certain yeasts. Representative bacterial probiotics are species of *Lactobacillus* [10–12], *Bifidobacterium* [13–15], *Bacillus* [16–18] and *Enterococcus* [19, 20]. Yeast probiotics include *Saccharomyces cerevisiae* [21] and *Saccharomyces boulardii* [22]. *Bacillus*, a genus of bacteria within the Phylum Bacillota, the Class Bacilli and the Order Bacillales, is a group of Gram‐positive bacteria which can be found in a broad range of different environments. Currently, over 400 *Bacillus* species (*Bacillus* spp.) have been validly published under the International Code of Nomenclature of Bacteria (ICNP) (https://www.bacterio.net/). *Bacillus* spp. are known for their ability to form resilient endospores, allowing them to survive in harsh environmental conditions, including the acidic environment of the stomach and the heat during food processing [23]. *Bacillus* spp. can produce a wide range of digestive enzymes, such as amylases, proteases and lipases, helping break down complex carbohydrates, proteins and fats in food [24]. Additionally, many *Bacillus* spp. are known for synthesising antimicrobial compounds, including bacteriocins, which combat harmful pathogens in the gut [25]. All these features make *Bacillus* spp. ideal candidates for probiotics.

**Figure 1:**
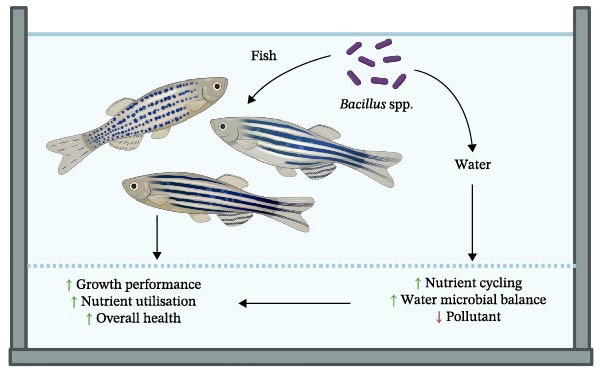
*Bacillus* spp. enhance fish growth performance by improving nutrient utilisation, promoting fish health and improving water quality. When *Bacillus* spp. are used as feed supplements, they not only enhance host nutrient utilisation and overall health but also improve water quality by promoting efficient nutrient cycling, maintaining microbial balance and reducing environmental pollutants. These improvements create a positive feedback loop that further supports fish health and performance within the aquaculture system. The figure was created by author Lu in BioRender (http://BioRender.com/467uuii).

The most frequently reported safe *Bacillus* spp. in fish farming are *B. subtilis*, *B. licheniformis*, *B. amyloliquefaciens*, *B. velezensis*, *B. pumilus*, *B. coagulans* and *B. megaterium*. This review provides a summary of recent applications of these seven *Bacillus* spp. in aquaculture, highlighting their roles in enhancing fish performance through improved nutrient digestion and absorption, promotion of fish health and improvement of water quality (Figure 1).

## 2. Methodology

### 2.1. A Glance of *Bacillus* spp. and Their Probiotic Applications in Finish Farming

**Figure 2:**
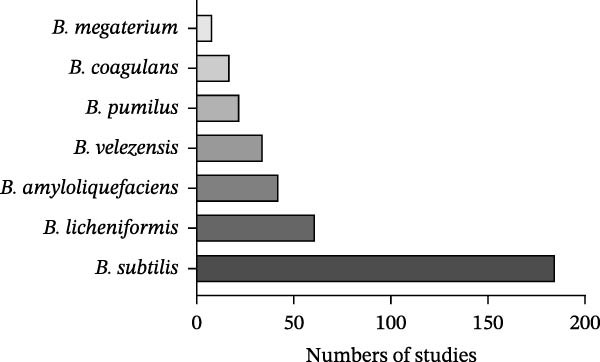
Distribution of *Bacillus* spp. used as probiotics in fish farming. A literature search on probiotic *Bacillus* in fish farming was conducted using the Scopus database to identify English‐language original research articles published between 2015 and 2024. The term “*Bacillus*” was searched within article titles, while “probiotic” and “fish,” “finfish,” “carp,” “catfish,” “cichlid,” “salmonid” or “milkfish” were searched within abstracts and keywords. A total of 412 research articles were found. Titles and abstracts were initially screened to determine their relevance to the study. Only research articles that aligned with the study objective were counted. The number of studies reporting each *Bacillus* specie was counted. The most frequently reported safe *Bacillus* spp. were *B. subtilis* (*n* = 185), *B. licheniformis* (*n* = 61), *B. amyloliquefaciens* (*n* = 42), *B. velezensis* (*n* = 34), *B. pumilus* (*n* = 22), *B. coagulans* (*n* = 17) and *B. megaterium* (*n* = 8).

To gain a good understanding of *Bacillus* spp. and their probiotic applications in finish farming, a literature search was first conducted on probiotic *Bacillus* in fish farming. Scopus, an abstract and citation database, was used to identify English original research articles. The term ‘*Bacillus*’ was used to search within the article title, while ‘probiotic’, and ‘fish’ or ‘finfish’ or ‘carp’ or ‘catfish’ or ‘cichlid’ or ‘salmonid’ or ‘milkfish’ were used to search within article abstract and keywords. A total of 412 research articles published between 2015 and 2024 were found in the database. Titles and abstracts were initially screened to determine their relevance to the study. Only research articles that aligned with the study objective were counted. The most frequently reported *Bacillus* spp. are *B. subtilis* (*n* = 185), *B. licheniformis* (*n* = 61), *B. amyloliquefaciens* (*n* = 42), *B. velezensis* (*n* = 34), *B. cereus* (*n* = 32), *B. pumilus* (*n* = 22), *B. coagulans* (*n* = 17) and *B. megaterium* (*n* = 8) (Figure 2). Among these eight *Bacillus* spp., seven are considered safe for use in animal feed, food or as sources of food and feed additives (AAFCO and EFSA). The exception is *B. cereus*, which is recognised as a pathogenic bacterium primarily due to its production of various toxins [26].

### 2.2. Inclusion and Exclusion Factors

To further refine the literature search, the keyword ‘growth’ was searched within the article titles, resulting in 106 research articles. These articles were screened individually according to the inclusion and exclusion criteria outlined in Table 1. Following this process, 59 studies met the eligibility criteria and were included in the present review.

**Table 1:** Inclusion and exclusion factors of database.

Inclusion factors
1. Studies evaluating safe and non‐pathogenic *Bacillus* strains 2. Use of a single *Bacillus* strain 3. Sufficient methodological detail to extract strain identity, fish species, trial duration, dietary dose and growth parameters (survival rate, specific growth rate and feed conversion ratio) 4. Dietary probiotic dose clearly reported or can be calculable as CFU/g feed

Exclusion factors
1. Studies using only mixed or multi‐strain *Bacillus* probiotic formulations 2. Absence of quantitative growth performance metrics 3. Use of heat‐inactivated probiotics (paraprobiotics)

## 3. *Bacillus* spp. in Enhancing Fish Growth Performance

Fish growth performance is an important indicator in fish farming, as higher growth performance means more productivity, profitability and sustainability. Growth performance is commonly evaluated using growth parameters and morphological indices. Frequently reported growth parameters include survival rate (SR), specific growth rate (SGR) and feed conversion ratio (FCR). Higher SGR reflects more efficient growth, while a lower FCR indicates improved feed utilisation efficiency [27]. In this review, growth metrics, including SR, SGR and FCR, were extracted from the 59 selected studies to evaluate the effects of *Bacillus* spp. on fish growth performance. Growth metrics (SR, SGR and FCR) are presented as percentage changes relative to the control group, in which fish were fed a basal diet without probiotics. Table S1 summarises the *Bacillus* spp., their isolation sources, fish species, trial durations, dietary doses and growth parameters reported across the 59 selected studies.

### 3.1. *Bacillus* spp. and Their Sources

**Figure 3:**
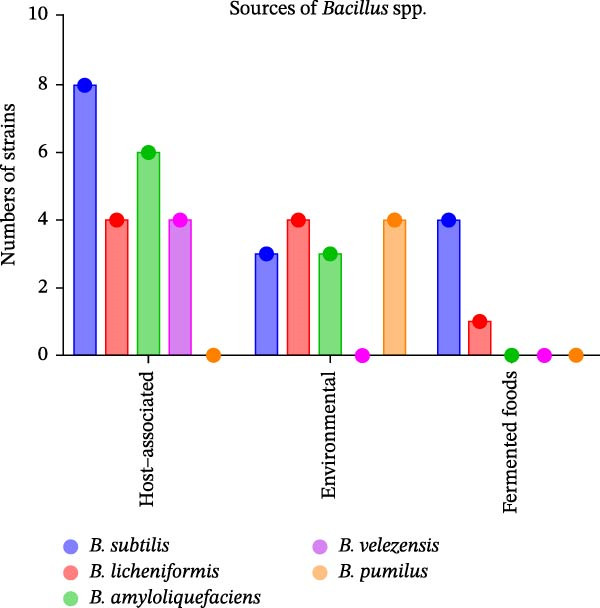
Sources of *Bacillus* spp. *Bacillus* strains were classified according to their reported origins as host‐associated (fish gut or intestine), environmental (soil, water, aquaculture environments and plant‐associated habitats) or fermented food–derived. Bars represent the number of distinct strains reported for each *Bacillus* species.

A total of seven *Bacillus* spp. were reported, with *B. subtilis* being the most frequently used species (*n* = 14), followed by *B. amyloliquefaciens* (*n* = 9), *B. licheniformis* (*n* = 8), *B. velezensis* (*n* = 4), *B. pumilus* (*n* = 2), *B. coagulans* (*n* = 1) and *B. megaterium* (*n* = 1). This distribution is consistent with the trend shown in Figure 2. These *Bacillus* spp. are primarily isolated from host‐associated sources (~42%), particularly the fish intestine and gut, followed by environmental sources (~17%) such as soil and aquaculture environments. A few strains originated from fermented foods (8%). In some studies, the isolation source was not reported, or commercial probiotic products were used without disclosure of strain origin, making source attribution unavailable. *Bacillus* strains were classified according to their reported origins as host‐associated, environmental or fermented food–derived (Figure 3). Among the species, *B. subtilis* shows the strongest host association, with the highest number of strains originating from fish tissues, followed by *B. amyloliquefaciens*. Approximately half of the *B. licheniformis* and *B. amyloliquefaciens* strains originated from host‐associated sources, while the remainder were isolated from the environment, indicating a more balanced distribution. In contrast, *B. velezensis* isolates were predominantly derived from host‐associated sources, whereas *B. pumilus* isolates were mainly derived from environmental sources. Fermented food–derived isolates are almost exclusively associated with *B. subtilis*, with only a single *B. amyloliquefaciens* strain identified from this source.

Collectively, the reviewed literature indicates that *B. subtilis* is the most studied probiotic species used in fish aquaculture. Across all *Bacillus* spp., host‐associated sources, particularly the fish gut and intestine, represent the primary origin of probiotic strains and may partly explain their reported effectiveness in enhancing fish growth performance.

### 3.2. Fish Species, Trial Duration and Probiotic Dosage

Approximately 30 fish species and hybrids, including both freshwater and marine species commonly used in aquaculture, were reported. Of the fish species included in Table S1, approximately two‐thirds were freshwater species, while one‐third were marine species. The most frequently studied species were Nile tilapia (*Oreochromis niloticus*), followed by hybrid grouper (*Epinephelus fuscoguttatus* × *E. lanceolatus*), rainbow trout (*Oncorhynchus mykiss*), rohu (*Labeo rohita*) and olive flounder (*Paralichthys olivaceus*), reflecting their importance in global aquaculture production.

The duration of the feeding trials varied widely, ranging from 20 to 300 days, with the majority of studies conducted for 42–60 days. Most trials lasted 56 or 60 days, which are the commonly used timeframes for assessing growth performance in aquaculture feeding experiments.

To ensure consistency and comparability, dietary doses were reported or recalculated as colony‐forming units per gram of feed (CFU/g) across all studies. The dietary inclusion levels of *Bacillus* spp. also varied among the reviewed studies, ranging from approximately 10–10^9^ CFU/g feed. Several studies evaluated multiple dose levels to assess dose–response effects. Statistically significant improvements in growth performance, particularly increases in SGR and reductions in FCR, were most observed at dietary doses of 10^6^–10^8^ CFU/g feed.

### 3.3. Effects of *Bacillus* spp. on Fish Growth Performance

SR was generally maintained or moderately improved, with no evidence of adverse effects associated with supplementation of *Bacillus* spp. The majority of trials reported significant increases in SGR (*p* ≤ 0.05) with *Bacillus* spp. supplemented groups compared with control groups. Only a small number of studies reported no change or non‐significant effects in SGR, which were often associated with short trial durations or non‐optimal doses. Improvements in SGR were frequently accompanied by reductions in FCR, indicating improved feed utilisation efficiency. Notably, significant improvements in SGR and FCR were most observed at dietary doses of 10^6^–10^8^ CFU/g feed, while lower doses produced more variable outcomes. Although a few studies reported better growth performance at higher doses, these effects were not consistent across fish species or *Bacillus* strains. Overall, these studies suggest that *Bacillus* spp. enhance growth performance through improved growth performance (SGR and FCR), while maintaining high SR.

**Figure 4:**
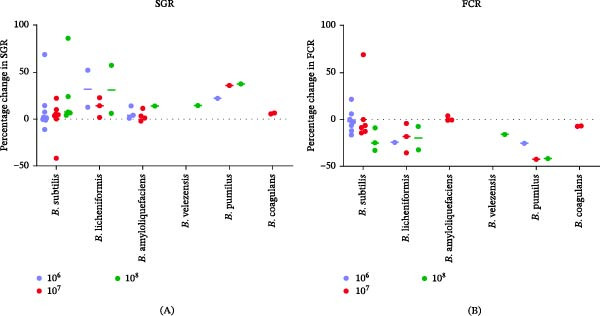
Effects of *Bacillus* spp. on fish growth performance. Analyses were conducted using the most commonly used probiotic doses (10^6^, 10^7^ and 10^8^ CFU/g) and trial durations (56 and 60 days). Growth performance was assessed as the percentage change in (A) specific growth rate (SGR) and (B) feed conversion ratio (FCR) relative to the control. Each point represents an individual study outcome, and the dashed horizontal line indicates the median values.

The most commonly used probiotic doses (10^6^, 10^7^ and 10^8^ CFU/g) and trial durations (56 and 60 days) were selected to evaluate the effects of *Bacillus* spp. on fish growth performance. Percentage changes in SGR and FCR relative to the control were used to assess the growth performance (Figure 4). Most data points are positive values, indicating a general enhancement of SGR following *Bacillus* supplementation. *B. subtilis* and *B. licheniformis* demonstrated consistent improvements in SGR, with several studies reporting increases exceeding 50%. Other *Bacillus* spp. also showed improved SGR, although the magnitude of the response and the number of available studies were lower (Figure 4A). Figure 4B shows an overall reduction in FCR (improved feed efficiency) following *Bacillus* supplementation. The greatest and most consistent improvements were observed in *B. pumilus*. *B. subtilis* and *B. amyloliquefaciens* also showed predominantly reduced FCR values, although with greater variability and a few positive outliers indicating worsened feed efficiency.

In summary, *Bacillus* spp. have been shown to significantly improve fish growth performance across a wide range of fish species. Host‐associated sources represent the most common origin of *Bacillus* spp. used in aquaculture research. Among the *Bacillus* spp. evaluated, *B. subtilis* consistently demonstrated the most pronounced and reproducible improvements in growth performance, particularly in SGR and FCR. Other *Bacillus* species, including *B. licheniformis* and *B. amyloliquefaciens*, also showed growth‐promoting potential, although the number of available studies remains comparatively limited. Based on these findings, the mechanisms by which *Bacillus* spp. enhance fish growth performance are discussed in the following section, using evidence from the 59 selected studies and other relevant literature.

## 4. Mechanisms of *Bacillus* spp. in Improving Fish Growth Performance

### 4.1. Spore‐Forming Stability in Gut Environments

*Bacillus* spp. are known to form endospores, which exhibit high stability during feed processing, storage and other environmental stressors. This spore‐forming capacity enables *Bacillus* spp. to withstand the harsh gastrointestinal (GI) environment, including gastric acidity and bile salts, and subsequently germinate into metabolically active cells in the intestine [23]. Following germination, these bacteria can contribute to improved growth performance by producing digestive enzymes, modulating the intestinal microbiota and enhancing nutrient utilisation and feed efficiency.

### 4.2. Enhancement of Nutrient Digestion and Absorption

Digestive enzymes play a crucial role in enhancing fish growth performance by facilitating the breakdown and absorption of nutrients. Enzymes such as amylase, protease and lipase facilitate the digestion of carbohydrates, proteins and fats, respectively, ensuring feed utilisation efficiency [28]. *Bacillus* spp. are particularly known for their capacity to produce and secrete these key beneficial enzymes [24], which can directly supplement endogenous digestive processes in fish and enhance overall digestive capacity.

Multiple studies have demonstrated that dietary supplementation with *Bacillus* spp. in various fish species results in concurrent enhancements in digestive enzyme activities and growth performance, which is reflected by increased SGR and reduced FCR (Table S2). For example, in striped catfish (*Pangasius hypophthalmus*), fish fed a diet supplemented with *B. subtilis* (10^8^ CFU/g) exhibited significantly increased intestinal amylase (9.2%), lipase (131.2%) and protease (79.9%) activities (*p*  < 0.05), which likely contributed to the observed improvements in SGR and FCR [29]. Similarly, dietary supplementation with *B. subtilis* (10^8^ CFU/g) in rainbow trout significantly increased intestinal trypsin (15%) and lipase (200%) activities (*p*  < 0.05), which was accompanied by enhanced growth performance [30]. Consistent results have also been reported in fish like hybrid grouper, Nile tilapia, tongue sole (*Cynoglossus semilaevisfed*), olive flounder, red sea bream (*Pagrus major*) and Pengze crucian carp (*Carassius auratus* var. Pengze), where *B. subtilis* supplementation led to significant improvements in both growth performance and intestinal digestive enzyme activities [31–37].

Other *Bacillus* species demonstrate similar mechanistic effects. *B. licheniformis* strains (10^5^–10^9^ CFU/g) showed similar enhancements in intestinal lipase (7.5%–120%), amylase (15.6%−133.3%) and protease (10.4%−59.8%) activities (*p*  < 0.05) in Nile tilapia and rohu [38–41]. Additionally, *B. amyloliquefaciens* and *B. velezensis* strains (10^6^–10^9^ CFU/g) further contributed to enhanced growth performance through the improvements in intestinal, gastric and serum protease/trypsin (8.9%–100%), amylase (5%−116.7%) and lipase (6.7%−114.3%) activities (*p*  < 0.05) in common carp (*Cyprinus carpio L*), hybrid grouper, yellow catfish (*Pelteobagrus fulvidraco*), rohu and Nile tilapia [31, 39, 42–45]. *B. megaterium* also demonstrated improved SGR and FCR, alongside with remarkable increases in protease (242%) and amylase (59%) activities (*p*  < 0.05) in catfish [46].

Overall, these findings suggest that *Bacillus* spp. enhance fish growth performance primarily through the upregulation of digestive enzyme activities, resulting in improved nutrient digestion, absorption and feed efficiency across a wide range of aquaculture species.

### 4.3. Promoting Health Benefits in Fish

#### 4.3.1. Gut Health Improvement

**Figure 5:**
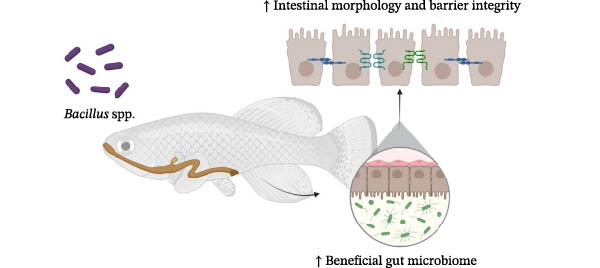
Gut health improvement mediated by *Bacillus* spp. in fish. Dietary *Bacillus* spp. modulate the gut microbiota, which subsequently enhances intestinal morphology and barrier integrity through strengthened epithelial structure and tight junction function, thereby supporting improved gut health and growth performance. The figure created in BioRender. Lu (2026) https://BioRender.com/iymfpoh.

The gut, commonly referred to as the GI tract, is a complex system. Its primary functions are the digestion of food, the absorption of essential nutrients and the excretion of waste products from the body [47]. Fish GI tract includes the mouth, oesophagus, stomach, intestines, pyloric caeca, liver, pancreas, rectum and anus for fish [48]. The structure of the GI tract varies across fish species, primarily based on diet. For example, some fish lack a stomach, relying instead on an extended intestine for digestion. Additionally, the presence and function of pyloric caeca, which aid in digestion and absorption, vary between species [49]. A healthy gut plays an important role in overall health and well‐being, as it facilitates the efficient processing of nutrients and supports the immune system. This review explores how *Bacillus* probiotics enhance gut health by promoting a balanced microbiota, enhancing intestinal structure and function, strengthening gut integrity through tight junction proteins, thereby contributing to enhanced fish growth performance (Figure 5).

##### 4.3.1.1. Modulation of Gut Microbiome

Gut microbiome refers to the diverse community of microorganisms colonising the GI tract [50]. Approximately 10^8^ bacterial cells per gram of intestinal content from more than 500 different species are found in the fish GI tract, with Proteobacteria, Firmicutes and Bacteroidetes making up 90% of the microbiota, alongside Fusobacteria, Actinobacteria and Verrucomicrobia [51, 52]. Gut microbiome balance means a stable and functionally diverse microbial community in which beneficial microorganisms predominate, while the opportunistic or pathogenic bacteria is restrained. In fish, maintaining microbiome balance is critical for optimising digestive efficiency, reinforcing intestinal barrier integrity, modulating immune responses and enhancing resistance to environmental stressors and pathogenic challenges [51, 53–55].

Dietary supplementation with *Bacillus* spp. has been shown to modulate gut microbiome composition, promoting a more balanced and functionally beneficial microbial community. *B. subtilis* strains at dietary doses ranging from approximately 10^4^–10^9^ CFU/g feed have been shown to significantly modulate gut microbial composition across multiple fish species, including largemouth bass, tongue sole, Japanese eel and hybrid grouper. Supplementation with *B. subtilis* was generally associated with the enrichment of beneficial or commensal microorganisms, including *Lactobacillus*, *Bifidobacterium*, *Bacillus*, *Cetobacterium*, *Romboutsia* and *Turicibacter*, while reducing the relative abundance of potentially harmful or opportunistic microorganisms, such as *Streptococcus pneumoniae*, *Plesiomonas*, *Staphylococcus*, *Mycoplasma* and *Achromobacter* [31, 32, 56–58]. Similar microbiome‐modulating effects have also been observed with other *Bacillus* spp., including *B. licheniformis*, *B. amyloliquefaciens* and *B. velezensis* [31, 43–45, 59], further supporting the role of *Bacillus* probiotics in improving gut microbiome balance in fish.

It was also suggested that the fish gut microbiota may influence overall fish performance not only through digestive and immune modulation but also via interactions with the central nervous system through the gut–brain axis. The gut microbiota produces metabolites in response to nutrients in the gut, which stimulate enteroendocrine cells in the GI tract and can also signal directly to the brain. These signals trigger the release of gut peptides that regulate appetite, feeding behaviour, food intake and energy homeostasis [60, 61]. Although direct mechanistic evidence for the gut–brain axis in fish remains limited, microbiome stabilisation induced by *Bacillus* probiotics may indirectly contribute to improved growth performance and resilience by optimising host metabolic and neurological functions. This represents an important area for future investigation.

##### 4.3.1.2. Modulation of Intestinal Morphology

Intestinal morphology refers to the structural features and organisation of the intestine, which are essential for its function in nutrient absorption, digestion and maintaining a barrier against pathogens. Studies have demonstrated that dietary supplementation with *Bacillus* can significantly enhance intestinal morphology across various fish species. In largemouth bass (*Micropterus salmoides*), dietary supplementation with *B. subtilis* (10^7^ CFU/g) resulted in a 30% increase in the length of intestinal villi (*p*  < 0.05), indicating improved surface area for nutrient absorption efficiency [56]. Similarly, continuous and discontinuous feeding olive flounder with *B. subtilis* (1.6 × 10^8^ CFU/g) led to 12% and 41% longer intestinal villi (*p*  < 0.05), and a 39% and 62% increase in stomach fold length (*p*  < 0.05), respectively, facilitating better nutrient uptake [62]. In Nile tilapia, diets containing *B. subtilis* (5.4 × 10^9^ CFU/g) improved intestinal morphology by elongating villi (19.5%, *p*  < 0.05) and thickening the submucosa layer (37.5%, *p*  < 0.05), thereby enhancing nutrient absorption and overall intestinal health [63]. Likewise, dietary supplementation of *B. pumilus* A1_YM_1 (10^6^, 10^7^ and 10^8^ CFU/g) significantly enhanced intestinal villi width, intestinal villi height, thicknesses of muscular layers, goblet cell number and microvillus height when compared to the control (*p*  < 0.05) in hybrid catfish [64], indicating improved intestinal development and mucosal function. Furthermore, *Bacillus* strains (*B. velezensis* PGSAK01, *B. stercoris* PGSAK05, *B. velezensis* PGSAK17 and *B. subtilis* PGSAK19 at 10^8^ CFU/g) isolated from hybrid grouper also significantly intestinal mucosal villus structure, characterised by a higher density of mucosal villi, enhancing nutrient absorption and gut health of hybrid groupers [65].

Overall, these findings demonstrated that dietary supplementation with *Bacillus* spp. enhances intestinal morphology, including increased villus height and density, reduced crypt depth and strengthened mucosal structure, thereby improving nutrient absorption efficiency and supporting enhanced fish growth performance.

##### 4.3.1.3. Regulation of Intestinal Barrier Integrity

Tight junction proteins are critical components of the intestinal barrier that regulate paracellular permeability and maintain a defence against harmful molecules, such as pathogens and toxins. These proteins, such as occludin, claudins and zonula occludens (ZO) proteins, form a seal between adjacent epithelial cells, contributing to the integrity and function of the intestinal epithelium [66]. *Bacillus* spp. supplementation has been shown to enhance intestinal barrier integrity through the upregulation of tight junction related genes in various fish species. For example, *B. subtilis* T20 was found to improve intestinal integrity by increasing intestinal villi height and upregulating gut barrier‐related genes (ZO‐1 and occludin) in common carp (*Cyprinus carpio*) (*p*  < 0.05) [67]. Similarly, dietary *B. subtilis* (10^9^ CFU/g) supplementation significantly increased the mRNA expression of ZO‐1, ZO‐2 and ZO‐3 compared with the control group (*p*  < 0.05) in grass carp (*Ctenopharyngodon idella*), indicating strengthened tight‐junction integrity [68].

Consistent effects have also been observed with other *Bacillus* strains. Supplementation with *B. licheniformis* FA6 (10^5^ and 10^6^ CFU/g) resulted in a significant (*p*  < 0.05) increase in the expression of ZO‐1, claudin c and occludin in grass carp [69]. Likewise, *B. velezensis* R‐71003 (10^8^ CFU/g) significantly upregulated the transcription of claudin and ZO‐1 (*p*  < 0.05) in the common carp, indicating enhanced tight‐junction function [45]. Supplementation of *Bacillus* spp. (*B. velezensis*, *B. subtilis* or *B. tequilensis* at 10^9^ CFU/g) significantly improved intestinal morphology and upregulated genes associated with intestinal tight junctions (occludin and ZO1) in hybrid grouper (*p*  < 0.05), enhancing gut barrier integrity in hybrid grouper [31]. Collectively, these findings demonstrate that *Bacillus* spp. enhance intestinal barrier integrity by upregulating tight junction–related genes, thereby supporting improved gut function and contributing to enhanced fish growth performance.

#### 4.3.2. Immunity Enhancement

##### 4.3.2.1. Stimulation of Innate Immune Responses

Innate immune responses, also known as non‐specific immunity, are the body’s first line of defence against infections or harmful stimuli. This rapid and broad response is activated within minutes to hours of encountering a pathogen or injury [70]. In fish studies, innate immune responses are commonly assessed using lysozyme activity, phagocytic activity, respiratory burst activity, complement activity and activities of acid phosphatase (ACP), alkaline phosphatase (AKP) and myeloperoxidase (MPO), with higher activities generally indicating stimulation or enhancement of innate immune responses [71–73]. In addition, the expression of innate immune‐related cytokines (e.g., IL‐1β, TNF‐α and IL‐10) provides molecular‐level insight into immune activation and regulation [74].

Dietary supplementation with *Bacillus* spp. has been consistently shown to stimulate innate immune responses in a wide range of aquaculture fish species (Table S3). Numerous studies report significant increases (*p*  < 0.05) in key innate immune biomarkers, including lysozyme activity, alternative complement components (C3 and C4), ACP, AKP and MPO activities following *Bacillus* supplementation. In parallel, several *Bacillus* strains modulate immune‐related gene expression, upregulating both pro‐inflammatory cytokines (e.g., IL‐1β and TNF‐α) and regulatory cytokines (e.g., IL‐10 and TGF‐β), thereby promoting effective immune activation while maintaining immune homeostasis.

##### 4.3.2.2. Suppression of Pathogenic Microorganisms

Innate immune enhancements are frequently associated with improved resistance to pathogenic infections [75], contributing to increased survival or reduced mortality following infection. Dietary supplementation with *Bacillus* spp. has been demonstrated to confer protection against a range of bacterial pathogens, including *Aeromonas*, *Vibrio*, *Streptococcus*, *Edwardsiella* and *Staphylococcus* species, which represent major bacterial pathogens in aquaculture systems (Table S3). Overall, by stimulating innate immune responses and suppressing pathogenic microorganisms, *Bacillus* spp. enhance fish immunity, thereby improving growth performance

### 4.4. Improving the Fish Farming Environment

In addition to host‐related benefits, the environmental effects of *Bacillus* spp. in aquaculture are necessary for consideration due to the potential impact of fish‐farming on the water ecosystem. *Bacillus* spp. probiotics play a beneficial role in protecting the water ecosystem and have environmental benefits through improvements in water quality. The improvement to water quality occurs through several mechanisms, including ensuring effective nutrient cycling, reducing impurities in the water like pathogenic microbes, pollutants and sediment accumulation, thereby allowing for a healthy aquatic environment for fish or other aquaculture species.

#### 4.4.1. Nutrient Cycling

*Bacillus* spp. contribute to nutrient cycling in aquaculture systems through the secrete extracellular enzymes to digest leftover food and animal matter [76–80]. In addition, they produce antioxidant and antibacterial hydrolysates, which help remove debris and maintain a cleaner aquatic environment [79]. Various *B. subtilis* strains exhibit the ability to hydrolyse fish feed components by producing extracellular enzymes such as protease, amylase, lipase and cellulase, enabling the breakdown of casein, starch, agar and cellulose in feed [78, 80–82]. Similarly, *B. velezensis* WLYS23 exhibits comparable enzyme production [77]. These enzymatic activities enable *Bacillus* spp. to participate in nutrient cycling by degrading and removing excess feed and organic matter from the water thereby reducing organic loading, improve water quality and supporting a healthier condition for fish.

The contribution of these enzymes to nutrient cycling depends on the administration strategy and probiotic dose. Direct feed inclusion is the most commonly used delivery strategy, in which probiotics are mixed with the basal diet [83], and this approach has been applied in most of the studies reviewed in the manuscript. When *Bacillus* spp. are delivered via direct feed inclusion, a substantial proportion of the supplemented feed is ingested by fish, where *Bacillus* spp. may germinate in the GI tract, contribute to feed degradation, and enhance fish growth performance [23]. Following digestion, a fraction of the probiotic cells and their extracellular enzymes may be released into the rearing water via faeces, enabling enzymatic activity. In addition, a proportion of the supplemented feed may persist uneaten in the rearing water, where extracellular enzymes can further contribute to nutrient cycling.

Although most studies report probiotic inclusion levels (CFU/g of feed or CFU/mL in water), quantitative distinction between the proportion of *Bacillus* spp. ingested by fish versus those persisting in the water is rarely reported. Enzymatic activities associated with these pathways can be assessed by analysing samples from the GI tract, faeces and rearing water. Nevertheless, both ingestion‐mediated and water‐mediated pathways allow *Bacillus* spp. to participate in nutrient cycling by reducing excess organic matter and facilitating the transformation and redistribution of nutrients within aquaculture systems.

#### 4.4.2. Water Microbial Balance Enhancement

*Bacillus* spp. improve the water quality by removing fungi and pathogenic bacteria while increasing the presence of healthy bacteria which promote fish health [79, 84–86]. *Fusarium* sp. is a fungus that causes swim bladder disease, a lethal fish illness that sporulates to spread [86]. In an examination of 34 *Bacillus* spp. isolated from samples of sludge pond water or the intestines of farmed *Pangasius*, it was determined that 20 of the strains were able to inhibit the spore activity of *Fusarium* sp., with a strain denoted BC11 having the highest antifungal inhibition [86]. This inhibition was reflected in another study where a combination of *B. megaterium* and *B. subtilis* (6 × 10^5^ CFU/mL) reduced the relative abundance of fungi (*Epicoccum* 98% *and Fusarium* 90%) in water sample from crucian carp farms [84].

Fish are susceptible to numerous pathogenic bacteria present in water, including *A. hydrophila* [87], *Cryptocaryon irritans* [88] and *Vibrio anguillarum* [89] which are responsible for infection, white‐spot disease and cold water vibriosis. *Bacillus* spp. demonstrate the ability to inhibit both gram‐positive and gram‐negative bacteria [35, 77, 79, 84]. Specifically, one of the most prevalently tested water pathogens, *A. hydrophila*, has been shown to be inhibited by a variety of *Bacillus* spp. including *B. subtilis* [78, 81, 90, 91], *B. velezenis* [91, 92], *B. pumilis* [91] and *B amyloliquefaciens* [90, 92–94], at concentrations of 10^8^–10^9^ CFU/mL [78]. *Bacillus* NP5 reduced the total amount of the *A. hydrophila* present in the water at concentrations of 10^4^ and 10^6^ CFU/mL [87]. This inhibitory effect extends to other pathogens like *C. irritans* [88], *V. anguillarum* [95–97], *Escherichia coli* [79, 82, 98], *Salmonella enteritidis* [79], *Aeromonas* spp. [77, 82], *Streptococcus* spp. [77, 98] and *Pseudomonas* spp [77, 84, 98, 99]. Another beneficial application of *Bacillus* spp. is the promotion of beneficial bacteria like *Proteobacteria*, *Actinobacteria*, *Comamonas* and *Stenotrophomonas*, which are nitrogen and phosphorus removing bacteria [84] and lactic acid bacteria, which assist in nutrient cycling [85].

#### 4.4.3. Pollutant Management

Heavy metal pollution is a major concern in fish farming, and *Bacillus* spp. have shown the ability to reduce and prevent metal bioaccumulation in fish [100–102]. In addition to heavy metals, water quality is also affected by excess nitrogenous compounds (such as ammonia, nitrate and nitrite) and phosphorus compounds [103–106], which are naturally present in water ecosystems. However, when these nutrients accumulate in excess, they can trigger algal blooms that lead to oxygen depletion and poor water quality [107, 108]. *Bacillus* spp. are able to improve water quality by modulating the levels of these compounds, thereby enhancing nutrient cycling and preventing eutrophication [107, 108]. Moreover, *Bacillus* spp. can degrade hydrocarbon pollutants, making them effective agents for comprehensive water purification in aquaculture environments [91]. Table 2 summarises the ability of *Bacillus* spp. in managing pollutants.

**Table 2:** *Bacillus* spp. in managing pollutants.

Pollutants	*Bacillus*	Effect on fish/water	References
Heavy metals	*B. coagulans* XY2	Protect zebrafish larvae against CuSO_4_	[102]
	*B. coagulans* SCC‐19	Prevent bioaccumulation of Cd^2+^ in common carp (*Cyprinus carpio* L.) kidney and liver	[100, 101]
	*B. subtilis* (selenium‐rich)	Reduce Hg accumulation and Hg‑induced inflammation and oxidative stress in common crap	[109–111]
Nitrogenous and phosphorus compounds	*B. subtilis*	TAN ↓53.1%, NO_2_^−^ ↓36.8% and NO_3_^−^ ↓15.6%	[35]
	*B. subtilis*	TAN ↓8.9% within 48 h	[112]
	*B. subtilis*	NH_3_ ↓41.2% within 120 h	[113]
	*B. subtilis* NP5	TAN ↓ and NO_2_^−^ ↓	[87, 114]
	*B. subtilis* NL110	NH_3_ ↓ and NO_3_^−^ ↓	[115]
	*B. licheniformis*	NH_3_ ↓ and NO_2_^−^ ↓	[104]
	*B. velezensis* LG37	NH_3_ ↓55.5% in a week	[116]
	*B. velezensis* AP193	Total N ↓43%, NO_3_^−^ ↓75% and total P ↓19%	[103]
	*B. subtilis* and *B. megaterium*	NH_3_ ↓46.3%, NO_2_^−^ ↓76.3%, NO_3_^−^ ↓35.6% and total P ↓80.3%	[84]
	*B. subtilis* and *B. licheniformis*	NH_3_ ↓, NO_2_^−^ ↓, total N ↓ and TAN ↓ Restore elevated NH_3_ concentrations to control levels	[85, 117–119]
	*B. subtilis*, *B. pumilus* and *B. licheniformis*	NH_3_ ↓58.2% and TAN ↓58.1%	[120]
Others	*B. subtilis* FS6 and *B. velezensis* FS26	Absorb xylene, chloroform and hydrocarbons.	[91]

Abbreviations: N, nitrogen; P, phosphorus; TAN, total ammonia nitrogen.

## 5. Conclusions and Future Directions

In conclusion, aquaculture plays a vital role in global food and nutrition security by providing a sustainable source of protein and essential nutrients [1], helping meet rising seafood demand while reducing pressure on wild fish stocks. Spore‐forming bacteria from the genus *Bacillus* are considered more suitable as probiotics in aquaculture than commonly used lactic acid bacteria, as they can better withstand harsh feed processing, environmental stress and storage conditions [23]. This review demonstrates that dietary supplementation of *Bacillus* spp. can improve fish growth performance by enhancing nutrient digestion and absorption, improving overall fish health and maintaining water quality in aquaculture systems.

Among all *Bacillus* spp, *B. subtilis* stands out as the most widely used species in fish farming, showing the highest number of applications across all benefit categories, including growth performance, health benefits and farming environment. This predominance is likely due to its well‐documented safety profile, high colonisation ability, the production of antimicrobial compounds, multifunctional benefits as described in this review and greater commercial availability [18, 121, 122]. In addition, *B. subtilis* appear to be the most abundant species isolated from diverse sources, including host‐associated (fish gut or intestine), environmental (soil, water, aquaculture environments and plant‐associated habitats) or fermented food–derived sources (Figure 3).

In contrast, other *Bacillus* spp. remain relatively understudied. *B. licheniformis*, *B. amyloliquefaciens* and *B. velezensis* show moderate representation, whereas, *B. pumilus*, *B. coagulans* and *B. megaterium* are reported far less frequently, suggesting limited application or investigation in fish farming (Figure 2). This disparity may reflect species‐specific limitations. For example, certain strains of *B. licheniformis* and *B. pumilus* have been reported to produce toxins [123, 124]. In addition, *B. velezensis* and *B. amyloliquefaciens* are closely related ‘operational group *B. amyloliquefaciens’* [125]. Their high genetic similarity leads to taxonomic confusion and regulatory challenges, complicating its development and use in probiotic product. *B. coagulans* is more commonly used as a human probiotic [126], whereas its suitability for aquatic environments remains underexplored and requires further investigation.

Despite these limitations, these underexplored *Bacillus* spp. represent promising opportunities for future research. They may possess unique enzymatic profiles, bioactive metabolites or immune‐modulating properties that are not present in *B. subtilis*. Moreover, certain strains may perform more effectively under specific environmental conditions or in particular fish species. In addition, this review excluded studies employing mixed or multi‐strain *Bacillus* probiotic formulations. In practice, combining *B. subtilis* with other *Bacillus* spp. may yield synergistic effects, enhancing probiotic efficacy through broader‐spectrum benefits. For instance, commercial products contain *B. subtilis* along with other *Bacillus* strains to maximise performance in aquaculture [85, 117–120]. Therefore, future research should prioritise the investigation of these *Bacillus* alternative species, assessing both their individual effects and their combined use with *B. subtilis*, as well as their integration with prebiotics and other health‐promoting additives, to further enhance aquaculture sustainability and productivity.

It was also notice that the mechanisms underlying the probiotic function of *Bacillus* spp. remains unclear at the metabolomic levels. While numerous studies have reported beneficial phenotypic outcomes, limited attention has been given to the specific metabolic pathways and bioactive metabolites mediating these effects. Multi‐omics approaches, including proteomics and metabolomics, could elucidate how *Bacillus* spp. modulate nutrient metabolism, immune function and disease resistance in fish, while also enabling the identification of key metabolic biomarkers associated with probiotic efficacy [127]. Such insights would support the rational selection of probiotic strains and formulations for more effective and sustainable aquaculture applications.

Lastly, when selecting novel *Bacillus* probiotics for aquaculture, key considerations include safety, host adaptability and the ability to effectively colonise the fish gut [128, 129]. Functional properties such as enzyme production, antimicrobial activity and immunomodulatory effects are also critical to ensuring the probiotic’s effectiveness and long‐term benefits in aquaculture systems [130]. Probiotics derived from marine and aquatic environments or fish gut microbiota can be ideal sources, offering competitive survival in aquatic conditions and high colonisation potential, respectively. For the *Bacillus* probiotics mentioned in this review, approximately 50% of them were derived from aquatic environments or fish gut microbiota, indicating a strong ecological adaptation of *Bacillus* strains to aquatic habitats and their potential as effective probiotics in aquaculture systems. Future efforts focused on exploring less‐studied species like *B. velezensis*, *B. pumilus*, *B. coagulans* and *B. megaterium* from the aquatic relevant sources may uncover novel probiotic functions and improve compatibility with fish hosts, thereby advancing the development of next‐generation probiotics for sustainable aquaculture.

## Author Contributions

**Yaoying Lu**: conceptualisation, writing, review, editing. **Natasha B. Bambridge:** writing, review. **Xiaojing Chen and Yunjiang Feng**: conceptualisation, review, editing, supervision.

## Acknowledgments

The authors acknowledge the financial support of the Marine Bioproducts Cooperative Research Centre (CRC), established and supported under the Australian Government’s CRC Program. The CRC Program supports industry‐led collaborations between industry, researchers and the community.

## Funding

This research was supported by the Marine Bioproducts Cooperative Research Centre (CRC) (Grant MBCRC319). Open access publishing facilitated by Griffith University, as part of the Wiley ‐ Griffith University agreement via the Council of Australasian University Librarians.

## Disclosure

All authors have read and agreed to the published version of the manuscript.

## Ethics Statement

This article is a review of previously published studies; therefore, ethical approval was not required.

## Conflicts of Interest

Author Xiaojing Chen is employed by Bioproton Pty Ltd. The other authors declare no conflicts of interest.

## Supporting Information

Additional supporting information can be found online in the Supporting Information section.

## Supporting information


**Supporting Information** The following supporting information is available for this manuscript: Table S1: Bacillus spp. in promoting fish growth performance; Table S2: Bacillus spp. in enhancing fish digestive enzyme activities; Table S3: Bacillus spp. in fish immunity enhancement.

## Data Availability

All data presented in this study are derived from previously published sources.
