# Supplementary material for: Bacillus spp. in Fish Farming: Enhancing Fish Performance, Health and Farming Practices
Source: Aquac Nutr. 2026 May 15;2026:3323307. doi: 10.1155/anu/3323307 (PMC13179295; doi:10.1155/anu/3323307)
Supplement: Supplementary file 1 — Supporting Information The following supporting information is available for this manuscript: Table S1: Bacillus spp. in promoting fish growth performance; Table S2: Bacillus spp. in enhancing fish digestive enzyme activities; Table S3: Bacillus spp. in fish immunity enhancement. [file ANU-2026-3323307-s001.docx]

Supplementary information for

*Bacillus* spp. in fish farming: enhancing fish performance, health and farming practices

Yaoying Lu ^a^, Natasha B. Bambridge ^a^, Xiaojing Chen ^b^ and Yunjiang Feng ^a, c, *^

^a^Institute for Biomedicine and Glycomics, Griffith University, Parklands Drive, Gold Coast, Queensland, 4222, Australia;

^b^Bioproton Pty Ltd., 55 Dulacca St, Brisbane, Queensland, 4110, Australia;

^c^School of Environment and Science, Griffith University, 170 Kessels Road, Brisbane, Queensland, 4111, Australia

***** Correspondence: y.feng@griffith.edu.au; Tel.: +61-(0)7-3735-8367

List of supporting information:

**Table S1:** *Bacillus* spp. in promoting fish growth performance……………………………...3

**Table S2:** *Bacillus* spp. in enhancing fish digestive enzyme activities……………………...11

**Table S3:** *Bacillus* spp. in fish immunity enhancement……………………..........................16

**References**………………………………………………………….………..........................20

Table S1: *Bacillus* spp. in promoting fish growth performance

| *Bacillus* | Sources of *Bacillus* | Fish | Trial (days) | Dose (CFU/g) | Performance metrics^1^ | | | References |
| --- | --- | --- | --- | --- | --- | --- | --- | --- |
|  |  |  |  |  | **SR** | **SGR** | **FCR** |  |
| *B. subtilis*  RT-BS07 | The gut of  healthy rainbow trout | Rainbow trout (*Oncorhynchus mykiss*) | 42 | 10^8^ | No change | ↑78.9% * | ↓9.8%* | [1] |
| *B. subtilis* | N/A | Largemouth bass (*Micropterus salmoides*) | 35 | 10^7^ | ↓2.5% | ↑3.0% * | N/A | [2] |
| *B. subtilis* natto NTU-18 | Fermented soybean (natto) | Japanese eel (*Anguilla japonica*) | 70 | 2.5 × 10^4^ | No change | ↑14.4% * | ↓13.8% * | [3] |
|  |  |  |  | 5 × 10^4^ | No change | ↑22.5% * | ↓23.1% * |  |
|  |  |  |  | 10^5^ | No change | ↑24.6% * | ↓25.6% * |  |
|  |  |  |  | 2 × 10^5^ | ↑1.27% | ↑15.5% * | ↓15.6% * |  |
| *B. subtilis* | N/A | Striped catfish (*Pangasius hypophthalmus*) | 56 | 10^6^ | N/A | ↑7.6% * | ↓16.5% * | [4] |
|  |  |  |  | 10^8^ | N/A | ↑85.1% * | ↓32.9% * |  |
|  |  |  |  | 10^10^ | N/A | ↑143.9% * | ↓65.1% * |  |
| *B. subtilis* | The intestinal tract of healthy darkbarbel catfish | Darkbarbel catfish (*Pelteobagrus fulvidraco*) | 56 | 10^4^ | ↓0.17% | ↑31.6% * | N/A | [5] |
|  |  |  |  | 10^5^ | ↓0.06% | ↑67.8% * | N/A |  |
|  |  |  |  | 10^6^ | ↓0.06% | ↑68.1%* | N/A |  |
| *B. subtilis* | Traditional Chinese fermented shrimp paste | Hybrid grouper (*Epinephelus fuscoguttatus* × *E. lanceolatus*) | 42 | 10^8^ | N/A | No change ^#^ | ↓20.2% * | [6, 7] |
| *B. subtilis* 6-3-1 | Intestines of hybrid grouper | Hybrid grouper | 42 | 10^8^ | N/A | ↑8.3% ^#^ | ↓38.3% * | [6, 7] |
| *B. subtilis* HAINUP40 | Natural pond water | Hybrid grouper | 42 | 10^8^ | N/A | ↑11.1% ^#^ | ↓35.1% * | [6, 7] |
| *B. subtilis* HAINUP40 | Natural pond water | Nile tilapia (*Oreochromis niloticus*) | 56 | 10^8^ | N/A | ↑24.0% * | ↓25.0% * | [8] |
| *B. subtilis* | N/A | Red tilapia (*Oreochromis mossambicus* × *O. niloticus*) | 70 | 2×10^6^ | ↑0.8% | ↑9.2% * | ↓12.64% * | [9] |
|  |  |  |  | 3×10^6^ | ↑2.0% | ↑15.3% * | ↓18.97% * |  |
|  |  |  |  | 4×10^6^ | ↓0.3% | ↑17.0% * | ↓21.26% * |  |
| *B. subtilis* | N/A | Thinlip Mullet (*Liza ramada*) | 56 | 10^6^ | ↑2.3% * | ↑14.4% * | ↓11.8% * | [10] |
| *B. subtilis* | N/A | Tongue sole  (*Cynoglossus semilaevisfed*) | 60 | 10^7^ | N/A | ↑22.2% * | ↓14.1% * | [11] |
| *B. subtilis* | N/A | Olive flounder (*Paralichthys olivaceus*) | ~300 | 10^8^ | ↑7.9% * | ↑4.1% * | N/A | [12] |
| *B. subtilis* | N/A | Grass carp (*Ctenopharyngodon idella*) | 28 | 10^9^ | ↑0.84% | ↑80.0% * | N/A | [13] |
| *B. subtilis* WB60 | The intestine of juvenile Japanese eel | Nile tilapia | 56 | 10^7^ | ↑7.4% | ↑4.6% | ↓9.0% ^#^ | [14] |
|  |  |  |  | 10^8^ | ↑5.6% | ↑7.6% * | ↓12.3% *^#^ |  |
| *B. subtilis* WB60 | The intestine of juvenile Japanese eel | Japanese eel | 56 | 10^6^ | ↑16.3%* | ↑0.8% ^#^ | ↓0.7% ^#^ | [15] |
|  |  |  |  | 10^7^ | ↑19.0%* | ↑3.2% ^#^ | ↓4.2% *^#^ |  |
|  |  |  |  | 10^8^ | ↑16.3%* | ↑4.0% ^#^ | ↓5.6% *^#^ |  |
| *B. subtilis* WB60 | The intestine of juvenile Japanese eel | Japanese eel | 56 | 5×10^6^ | ↓2.7% | ↑1.4% | ↓1.7% ^#^ | [16] |
|  |  |  |  | 10^7^ | ↓2.7% | ↑5% * | ↓5.6% *^#^ |  |
| *B. subtilis* C-3102 | Soil | Hybrid surubim (*Pseudoplatystoma reticulatum* × *P. corruscans*) | 20 | 8.4×10^6^ | N/A | ↑46.7% | ↓31.5% | [17] |
|  |  |  |  | 1.7×10^7^ | N/A | ↑53.3% | ↓33.5% |  |
|  |  |  |  | 3.3×10^7^ | N/A | ↑86.7% | ↓42.9% |  |
|  |  |  |  | 3.5×10^7^ | N/A | ↑73.3% | ↓36.6% |  |
| *B. subtilis* C-3102 | Soil | *Pseudoplatystoma reticulatum* | 60 | 2×10⁷ | ↓8.5% | ↑4.7% * | ↓12.7% | [18] |
| *B. subtilis* C-3102 | Soil | Red sea bream (*Pagrus major*) | 60 | 10^1^ | ↑10.0% | ↑7.1% * | ↓7.5% ^#^ | [19] |
|  |  |  |  | 10^3^ | ↑6.6% | ↑9.7% * | ↓2.5% ^#^ |  |
|  |  |  |  | 10^5^ | ↑10.0% | ↑10.9% * | ↓16.3% *^#^ |  |
|  |  |  |  | 10^7^ | ↑8.5% | ↑10.1% * | ↓8.8% ^#^ |  |
| *B. subtilis* C-3102 | Soil | Red sea bream | 30 | 4.88×10^9^ | N/A | ↑6.0% | ↓3.8% ^#^ | [20] |
|  |  |  | 60 |  | ↑3.6% | ↑7.3%* | ↓5.6% ^#^ |  |
| *B. subtilis* | N/A | Nile tilapia | 50 | 1.19×10^5^ | No change | ↑7.6% * | ↓0.7% | [21] |
|  |  |  |  | 2.38×10^5^ | No change | ↑12.5% * | ↓1.9% * |  |
|  |  |  |  | 3.57×10^5^ | No change | ↑18.9% * | ↓4.5% * |  |
|  |  |  |  | 4.76×10^5^ | No change | ↑24.5% * | ↓4.5% * |  |
| *B. subtilis* | Intestinal microbiota of olive flounder | Olive flounder | 84 | 10^7^ | ↑4.1% | ↑2.9% | ↓8.5% | [22] |
|  |  |  |  | 3×10^7^ | ↓2.0% | ↑5.8% | ↓7.7% |  |
|  |  |  |  | 5×10^7^ | ↑3.1% | ↑10.1% * | ↓16.9% * |  |
| *B. subtilis* | N/A | Pengze crucian carp (*Carassius auratus* var. Pengze) | 70 | 10^6^ | N/A | ↑4.4% * | ↓10.6% ^#^ | [23] |
| *B. subtilis* E20 | Natto | Parrot fish (*Oplegnathus fasciatus*) | 56 | 10^5^ | ↓7.7% | ↑20.0%^#^ | ↓20.2% ^#^ | [24] |
|  |  |  |  | 10^6^ | ↓5.8% | ↓11.2%^#^ | ↑20.3% *^#^ |  |
|  |  |  |  | 10^7^ | ↓3.9% | ↓41.9%^#^ | ↑64.9% *^#^ |  |
| *B. subtilis* | N/A | Tambaqui (*Colossoma macropomum*) | 90 | 2×10^6^ | No change | N/A | ↓20.2% | [25] |
| *B. subtilis* GPSAK9 | The intestine of hybrid grouper | Hybrid grouper | 42 | 10^9^ | ↓6.8% | ↑7.5% * | ↓15.1% | [26] |
| *B. subtilis* TPS4 | The gut of Nile tilapia | Nile tilapia | 28 | 8×10^6^ | N/A | ↑25.2% * | ↓24.3% * | [27] |
| *B. subtilis* MRS11 | The intestinal mucus of Nile tilapia | Nile tilapia | 60 | 10^6^ | No change | ↑5.8% * | ↓6.3% * | [28] |
|  |  |  |  | 10⁸ | No change | ↑6.5% * | ↓9.0% * |  |
| *B. subtilis* DSM 32315 | N/A | Nile tilapia | 56 | 2×10^6^ | ↑1.7% | ↑2.2% * | ↓2.3% | [29] |
|  |  |  |  | 4×10^6^ | ↓2.8% | ↓0.4% | ↑6.0% * |  |
|  |  |  |  | 6×10^6^ | ↓1.7% | ↓1.2% | No change |  |
| *B. subtilis* DSM 32315 | N/A | Largemouth bass | 63 | 3 × 10^6^ | N/A | ↑1.6% | ↑1.8% | [30] |
|  |  |  |  | 6 × 10^6^ | N/A | No change | ↑0.9% |  |
| *B. subtilis* SB3086 | N/A | Nile tilapia | 21 | 8.5×10^7^ | N/A | ↓14.3% | ↓0.7% | [31] |
| *B. subtilis* SB3295 | N/A | Nile tilapia | 21 | 7.3×10^7^ | N/A | ↑2.9% | ↑8.5% | [31] |
| *B. subtilis* SB3615 | N/A | Nile tilapia | 21 | 8.2×10^7^ | N/A | ↓2.9% | No change | [31] |
| *B. subtilis* | N/A | Nile tilapia | 56 | 4.75×10^7^ | N/A | ↑0.3% | No change | [32] |
| *B. licheniformis*  KU556167 | The gut of Rohu | Rohu (*Labeo rohita*) | 60 | 10^7^ | N/A | ↑22.8% * | ↓18.1% * | [33] |
| *B. licheniformis* KU556167 | The gut of Rohu | Rohu | 90 | 10^7^ | 3.6% | ↑12.6% * | ↓16.7% * | [34] |
| *B. licheniformis* SB3086 | Soil | Nile tilapia | 56 | 10^5^ | ↑10.3% * | ↑9.5% | ↓7.1% * | [35] |
|  |  |  |  | 10^7^ | ↑13.6% * | ↑14.3% * | ↓35.7% * |  |
|  |  |  |  | 10^9^ | ↑23.1% * | ↑19.0% * | ↓50.0% * |  |
| *B. licheniformis* FA6 | The intestinal of grass carp | Grass carp | 56 | 10^5^ | N/A | ↑10.2% * | N/A | [36] |
|  |  |  |  | 10^6^ | N/A | ↑12.7% * | N/A |  |
| *B. licheniformis* HGA8B | The gastrointestinal tract of *Anabas testudineus* | Nile tilapia | 60 | 10^6^ | N/A | ↑51.2% * | ↓24.4% * | [37] |
|  |  |  |  | 10^8^ | N/A | ↑56.0% * | ↓32.5% * |  |
| *B. licheniformis* Dahb1 | The shrimp culture environment | Nile tilapia | 28 | 10^5^ | N/A | ↑73.8% * | ↓25.6% * | [38] |
|  |  |  |  | 10^7^ | N/A | ↑106.7% * | ↓29.6% * |  |
| *B. licheniformis* | N/A | Nile tilapia | 70 | 4×10^6^ | ↑1.1% | ↓0.3% | ↓1.5% | [39] |
|  |  |  |  | 8×10^6^ | ↑2.2% | ↑2.6% * | ↓3.8% |  |
|  |  |  |  | 1.2×10^7^ | ↓1.1% | ↑4.0% * | ↓3.8% |  |
|  |  |  |  | 1.6×10^7^ | No change | ↑2.6% * | ↓3.1% |  |
|  |  |  |  | 2.0×10^7^ | ↓3.5% | ↑4.0% * | ↓2.3% |  |
| *B. licheniformis* ATCC 11946 | N/A | Northern whiting (*Sillago sihama* Forssk´al) | 56 | 10^9^ | ↑20.5% * | ↑16.5% * | ↓33.6% * | [40] |
| *B. licheniformis* ATCC 12759 | A cassava tuber plant | Rainbow trout | 60 | 2.2 × 10^7^ | No change | ↑1.6% * | ↓4.2% * | [41] |
| *B. licheniformis* IBRC-M 10204 | N/A | Goldfish (*Carassius auratus*) | 56 | 10^8^ | No change | ↑6.2% | ↓7.5% | [42] |
| *B. amyloliquefaciens*  KU556165 | The gut of Rohu | Rohu | 60 | 10⁷ | N/A | ↑3.3% * | ↓3.0% * | [33] |
| *B. amyloliquefaciens* AV5 | The intestines of Nile tilapia | Nile tilapia | 30 | 10^6^ | ↑0.5% | ↑18.3% | ↓13.6% | [43] |
|  |  |  |  | 10^8^ | ↑1.8% | ↑45.8%* | ↓43.2%* |  |
| *B. amyloliquefaciens* LSG2-8 | The intestines of *Rhynchocypris lagowskii* | *Rhynchocypris lagowskii* | 56 | 10^6^ | N/A | ↑4.1% | ↓19.4% ^#^* | [44, 45] |
|  |  |  |  | 10^7^ | N/A | ↑11.6% * | ↓26.4% ^#^* |  |
|  |  |  |  | 10^8^ | N/A | ↑14.0% * | ↓29.3% ^#^* |  |
|  |  |  |  | 10^9^ | N/A | ↑11.6% * | ↓20.0% ^#^* |  |
| *B. amyloliquefaciens* | The intestine of yellow catfish | Yellow catfish (*Pelteobagrus fulvidraco*) | 28 | 10^6^ | N/A | ↑40.0% * | ↓14.8 * | [46] |
| *B. amyloliquefaciens* GB-9 | The intestinal tract of the white spotted bamboo shark (*Chiloscyllium plagiosum*) | Hybrid sturgeon (*Acipenser schrenkii* × *A. baeri*) | 28 | 10^7^ | ↑6.7% | ↑2.4% * | ↓4.3% * | [47, 48] |
| *B. amyloliquefaciens* JFP2 | Fermented seafood | Rock bream (*Oplegnathus fasciatus*) | 70 | 1.4×10^6^ | N/A | ↑15.3% * | ↓14.7% * | [49] |
| *B. amyloliquefaciens* TPS17 | The gut of Nile tilapia | Nile tilapia | 28 | 8×10^6^ | N/A | ↑15.4% | ↓16.2% * | [27] |
| *B. amyloliquefaciens* B-1895 | Soil | Rainbow trout | 45 | 6×10^7^ | ↑2.1% | ↑29.6% ^#^ | ↑25.0% ^#^ | [50, 51] |
| *B. amyloliquefaciens* | The intestine of Nile tilapia | Nile tilapia | 56 | 10^7^ | ↑0.9% | ↑1.1% | ↑3.8% | [52] |
| *B. amyloliquefaciens* CECT 5940 | Soil | Nile tilapia | 60 | 10^5^ | ↑5.6% | ↑11.6% * | ↓18.3% * | [53, 54] |
| *B. amyloliquefaciens* CECT 5940 | Soil | Nile tilapia | 60 | 1×10^5^ | N/A | ↑3.6% | ↓9.4% | [55] |
|  |  |  |  | 2×10^5^ | N/A | ↑2.3% | ↓4.7% |  |
| *B. amyloliquefaciens* CECT 5940 | Soil | Sea bream (*Sparus aurata*) | 100 | 10^6^ | N/A | ↓1.9% | ↓2.5% ^#^ | [56] |
| *B. amyloliquefaciens* CECT 5940 | Soil | Nile tilapia | 90 | 1 × 10^6^ | N/A | ↓0.9% | ↓2.5% ^#^ | [57] |
|  |  |  |  | 5 × 10^6^ | N/A | ↑2.7% | ↑6.3% ^#^ |  |
|  |  |  |  | 1 × 10^7^ | N/A | ↑7.3% | ↓7.4% ^#^ |  |
| *B. amyloliquefaciens* AP193 | Soybean rhizosphere | Nile tilapia | 21 | 7.7×10^7^ | N/A | ↓5.7% | ↓4.9% | [31] |
| *B. amyloliquefaciens* AP193 | Soybean rhizosphere | Nile tilapia | 56 | 5.5×10^7^ | N/A | ↓1.9% ^#^ | ↓0.8% | [32] |
| *B. velezensis* GPSAK4 | The intestine of hybrid grouper | Hybrid grouper | 42 | 10^9^ | ↓2.3% | ↑7.1% * | ↓15.1% | [26] |
| *B. velezensis* LF01 | Nile tilapia | Nile tilapia | 63 | 10^9^ | ↑2.6% | ↑7.3%* | N/A | [58] |
| *B. velezensis* TPS3N | The gut of Nile tilapia | Nile tilapia | 28 | 8×10^6^ | N/A | ↑18.9% | ↓18.4% * | [27] |
| *B. velezensis* R7–1003 | The intestinal tract of the common carp | Common carp (*Cyprinus carpio L*.) | 60 | 10^8^ | N/A | ↑14.6% * | ↓15.9% * | [59] |
| *B. pumilus* A1_YM_1 | Biofertilizer | Hybrid catfish (*Clarias macrocephalus* × *C. gariepinus*) | 56 | 10^6^ | ↓2.3% | ↑22.2% | ↓25.4% * | [60] |
|  |  |  |  | 10^7^ | ↓2.3% | ↑35.9% * | ↓42.4% * |  |
|  |  |  |  | 10^8^ | No change | ↑37.6% * | ↓41.8% * |  |
| *B. pumilus* 1486^T^ | N/A | Nile tilapia | 84 | 1.85×10^2^ | N/A | ↑6.5% * | ↓9.6% * | [61] |
| *B. coagulans* ATCC 7050 | Evaporated milk | Northern whiting | 56 | 10^9^ | ↑12.2% | ↑14.4% * | ↓35.4% * | [40] |
| *B. coagulans* | N/A | Gibel carp (*Carassius auratus gibelio*) | 56 | 10^7^ | N/A | ↑6.5% * | ↓7.3% | [62] |
|  |  |  |  | 2×10^7^ | N/A | ↑5.6% | ↓6.9% |  |
| *B. megaterium* PTB 1.4 | The gut of the catfish | Catfish (*Clarias* sp.) | 30 | 1.2×10^7^ | No change | ↑35.9% * | ↓29.5% * | [63] |

**Note:^1^ Growth metrics (SR, SGR and FCR) are presented as percentage changes relative to the control group, in which fish were fed a basal diet without probiotics. Survival rate (SR, %) = (Final number of fish)/(Initial number of fish)×100; Specific growth rate (SGR, %) = (In (final weight)- In(initial weight))/Days×100; Feed conversion ratio (FCR) = (Feed intake)/(Weight gain); N/A indicates data not available; *** **indicates statistically significant differences compared with the control group (P ≤ 0.05); # indicates values calculated by the authors based on information provided in the original manuscript.**

Table S2: *Bacillus* spp. in enhancing fish digestive enzyme activities

| *Bacillus* | Fish species | Dose (CFU/g) | Sample location | Digestive enzymes | Activities compared to control^2^ | References |
| --- | --- | --- | --- | --- | --- | --- |
| *B. subtilis* | Rainbow trout | 10^8^ | Intestine | Amylase | No significant change | [1] |
|  |  |  |  | Lipase | ↑200% * |  |
|  |  |  |  | Trypsin | ↑15% * |  |
| *B. subtilis* | Striped catfish | 10^6^ | Intestine | Amylase | ↑2% * | [4] |
|  |  |  |  | Lipase | ↓78.20% * |  |
|  |  |  |  | Protease | ↑58.9% * |  |
|  |  | 10^8^ | Intestine | Amylase | ↑9.2% * |  |
|  |  |  |  | Lipase | ↑131.2% * |  |
|  |  |  |  | Protease | ↑79.9% * |  |
|  |  | 10^10^ | Intestine | Amylase | ↑46.1% * |  |
|  |  |  |  | Lipase | ↓33.50% * |  |
|  |  |  |  | Protease | ↑157.8% * |  |
| *B. subtilis* | Hybrid grouper | 10^8^ | Intestine | Pepsin | No significant change | [6] |
|  |  |  |  | Trypsin | No significant change |  |
|  |  |  |  | Lipase | ↑86% * |  |
| *B. subtilis* 6-3-1 | Hybrid grouper | 10^8^ | Intestine | Pepsin | ↑30% * |  |
|  |  |  |  | Trypsin | No significant change |  |
|  |  |  |  | Lipase | No significant change |  |
| *B. subtilis* HAINUP40 | Hybrid grouper | 10^8^ | Intestine | Pepsin | ↑13% * |  |
|  |  |  |  | Trypsin | ↑19% * |  |
|  |  |  |  | Lipase | No significant change |  |
| *B. subtilis* HAINUP40 | Nile tilapia | 10^8^ | Intestine | Amylase | ↑186% * | [8] |
|  |  |  |  | Protease | ↑192% * |  |
| *B. subtilis* | Tongue sole | 10^7^ | Intestine, foregut | Protease | ↑80.7% * | [11] |
|  |  |  |  | Amylase | ↑50% * |  |
|  |  |  |  | Lipase | ↑23.7% * |  |
|  |  | 10^7^ | Intestine, midgut | Protease | ↑16.2% * |  |
|  |  |  |  | Amylase | ↑18.9% * |  |
|  |  |  |  | Lipase | ↑20.7% * |  |
|  |  | 10^7^ | Intestine, hindgut | Protease | ↑38.8% * |  |
|  |  |  |  | Amylase | No significant change |  |
|  |  |  |  | Lipase | ↑63.6% * |  |
| *B. subtilis* | Olive flounder | 10^8^ | Liver | Protease | ↑109% * | [12] |
|  |  |  |  | Amylase | No significant change |  |
|  |  |  |  | Lipase | ↑36.8% * |  |
|  |  | 10^8^ | Stomach | Protease | ↑54% * |  |
|  |  |  |  | Amylase | ↑38.9% * |  |
|  |  |  |  | Lipase | ↑76.3% * |  |
|  |  | 10^8^ | Intestine | Protease | ↑63% * |  |
|  |  |  |  | Amylase | No significant change |  |
|  |  |  |  | Lipase | ↑36.9% * |  |
| *B. subtilis* WB60 | Nile tilapia | 10^8^ | Not specified | Trypsin | ↑78.1% * | [14] |
| *B. subtilis* C-3102 | Red sea bream | 10^1^ | Digestive tract | Protease | ↑9% * | [19] |
|  |  |  |  | Amylase | ↑15% |  |
|  |  |  |  | Lipase | ↑7.90% |  |
|  |  | 10^3^ | Digestive tract | Protease | ↑16% * |  |
|  |  |  |  | Amylase | ↑20% |  |
|  |  |  |  | Lipase | ↑7.30% |  |
|  |  | 10^5^ | Digestive tract | Protease | ↑9% * |  |
|  |  |  |  | Amylase | ↑30% * |  |
|  |  |  |  | Lipase | ↑10.9% * |  |
|  |  | 10^7^ | Digestive tract | Protease | ↑31% * |  |
|  |  |  |  | Amylase | ↑25% |  |
|  |  |  |  | Lipase | ↑3% |  |
| *B. subtilis* C-3102 | Red sea bream | 4.88×10^9^ | Digestive tract | Protease | ↑55.6% * | [20] |
|  |  |  |  | Amylase | ↑42.0% * |  |
| *B. subtilis* | Nile tilapia | 1.19×10^5^ | Intestine | Protease | ↓0.50% | [21] |
|  |  |  |  | Lipase | ↑24.7% * |  |
|  |  | 2.38×10^5^ | Intestine | Protease | ↑3.3% * |  |
|  |  |  |  | Lipase | ↑36% * |  |
|  |  | 3.57×10**^5^** | Intestine | Protease | ↑12.7% * |  |
|  |  |  |  | Lipase | ↑49% * |  |
|  |  | 4.76×10**^5^** | Intestine | Protease | ↑10.4% * |  |
|  |  |  |  | Lipase | ↑40% * |  |
| *B. subtilis* | Pengze crucian carp | 10^6^ | Intestine | Amylase | ↑40.8% * | [23] |
|  |  |  |  | Lipase | ↑35.9% * |  |
|  |  |  |  | Trypsin | ↑164.9% * |  |
| *B. subtilis* GPSAK9 | Hybrid grouper | 10^9^ | Intestine | Trypsin | ↑0.80% | [26] |
|  |  |  |  | Lipase | ↑63% * |  |
|  |  |  |  | Amylase | ↑16% |  |
| *B. subtilis* TPS4 | Nile tilapia | 8×10^6^ | Serum | Trypsin | ↑76.2% * | [27] |
|  |  |  |  | Lipase | ↑96.4% * |  |
| *B. licheniformis* KU556167 | Rohu | 10^7^ | Intestine | Amylase | ↑15.6% * | [33] |
|  |  |  |  | Protease | ↑10.4% * |  |
|  |  |  |  | Lipase | ↑7.5% * |  |
| *B. licheniformis* KU556167 | Rohu | 10^7^ | GI tract | Amylase | ↑25% * | [34] |
|  |  |  |  | Protease | ↑20% * |  |
|  |  |  |  | Lipase | ↑18% * |  |
| *B. licheniformis* SB3086 | Nile tilapia | 10^5^ | Intestine | Protease | ↑22.7% * | [35] |
|  |  |  |  | Lipase | ↑40% * |  |
|  |  |  |  | Amylase | ↑46.7% * |  |
|  |  | 10^7^ | Intestine | Protease | ↑34% * |  |
|  |  |  |  | Lipase | ↑84% * |  |
|  |  |  |  | Amylase | ↑80% * |  |
|  |  | 10^9^ | Intestine | Protease | ↑59.8% * |  |
|  |  |  |  | Lipase | ↑120% * |  |
|  |  |  |  | Amylase | ↑133.3% * |  |
| *B. licheniformis* HGA8B | Nile tilapia | 10^6^ | Intestine | Protease | ↑42.4% * | [37] |
|  |  |  |  | Lipase | ↑55% * |  |
|  |  |  |  | Amylase | ↑65.3% * |  |
|  |  | 10^8^ | Intestine | Protease | ↑48% * |  |
|  |  |  |  | Lipase | ↑56% * |  |
|  |  |  |  | Amylase | ↑68.6% * |  |
| *B. amyloliquefaciens* TPS17 | Nile tilapia | 8×10^6^ | Serum | Amylase | No significant change | [27] |
|  |  |  |  | Trypsin | ↑85.7% * |  |
|  |  |  |  | Lipase | ↑100% * |  |
| *B. amyloliquefaciens* KU556165 | Rohu | 10^7^ | Intestine | Amylase | ↑17.3% * | [33] |
|  |  |  |  | Protease | ↑10.1% * |  |
|  |  |  |  | Lipase | ↑6.7% * |  |
| *B. amyloliquefaciens* | Yellow catfish | 10^6^ | Intestine | Amylase | ↑116.7% * | [46] |
|  |  |  |  | Protease | ↑71.4% * |  |
| *B. amyloliquefaciens* | Nile tilapia | 10^7^ | Foregut | Lipase | ↑336% * |  |
|  |  |  |  | Amylase | ↑91% * | [52] |
|  |  |  | Hindgut | Lipase | ↑43% * |  |
|  |  |  |  | Amylase | ↑5% * |  |
|  |  |  | Stomach | Protease | ↑17% * |  |
| *B. velezensis* GPSAK4 | Hybrid grouper | 10^9^ | Intestine | Trypsin | ↑8.90% | [26] |
|  |  |  |  | Lipase | ↑39.9% * |  |
|  |  |  |  | Amylase | ↑58.9% * |  |
| *B. velezensis* TPS3N | Nile tilapia | 8×10^6^ | Serum | Amylase | No significant change | [27] |
|  |  |  |  | Trypsin | ↑100% * |  |
|  |  |  |  | Lipase | ↑114.3% * |  |
| *B. velezensis* R-71003 | Common carp | 10^8^ | Intestine | Amylase | ↑14% * | [59] |
|  |  |  |  | Protease | ↑14% * |  |
| *B. megaterium* PTB 1.4 | Catfish | 1.2×10^7^ | Digestive tract | Protease | ↑242% * | [63] |
|  |  |  |  | Amylase | ↑59% * |  |

**Note: ^2^Digestive enzyme activities are presented as percentage changes relative to the control group, in which fish were fed a basal diet without probiotics. *** **indicates statistically significant differences compared with the control group (P ≤ 0.05).**

Table S3: *Bacillus* spp. in fish immunity enhancement

| *Bacillus* | Fish species | Dose (CFU/g) | Innate immune response (P<0.05) | Response to pathogen challenge (P<0.05) | References |
| --- | --- | --- | --- | --- | --- |
| *B. subtilis*  RT-BS07 | Rainbow trout | 10^8^ | ↑ IL-1β expression (intestine, muscle);  ↑ AKP activity (liver, spleen, intestine) | ↑ Survival (+75%) at day 12 post *Aeromonas hydrophila* challenge (significance not reported) | [1] |
| *B. subtilis* | Largemouth bass | 10^8^ | ↑AKP activity (intestine, serum) | N/A | [2] |
| *B. subtilis* natto NTU-18 | Japanese eel | 10^5^; 2×10^5^ | ↑ IgM and HSP70 expression (liver, head kidney) | ↑ Survival (+202% and +166%) at day 20 post *Edwardsiella tarda* challenge | [3] |
| *B. subtilis* | Darkbarbel catfish | 10^4^; 10^5^; 10^6^ | ↑ Lysozyme and AKP activities (plasma); ↑ IL-10, TGF-β, IgM, C3 expression (kidney) | ↓ Cumulative molarity (-18%, -34% and -38%) at day 7 post *A. hydrophila* challenge | [5] |
| *B. subtilis* MRS11 | Nile tilapia | 10^6^; 10^8^ | ↑ TGF-β, IL-10, IL-1β expression (intestine) | ↑Survival (+130% and 140%) at day 21 post *Streptococcus iniae* challenge | [28] |
| *B. subtilis* DSM 32315 | Nile tilapia | 2×10^6^ | ↑ Lysozyme activity (plasma), C3, IL-10 expression (liver) | N/A | [29] |
| *B. subtilis* DSM 32315 | Largemouth bass | 3×10^6^ | ↑ IgM and C4 levels (serum); ↓ IL-1β, IL-8, TNF-α and TGF-β1 expression (intestine) | N/A | [30] |
|  |  | 6×10^6^ | ↑ Lysozyme, C3 levels (serum) |  |  |
| *B. subtilis* | Grass carp | 10^9^ | ↑ C3, C4 levels and AKP activity (serum) | N/A | [13] |
| *B. subtilis* WB60 | Nile tilapia | 10^8^ | ↑ Lysozyme and MPO activities (serum); ↑ IL-1β, IFN-γ, TNF-α expression (intestine) | ↑ Survival (+100% and +140%) at day 13 post *A. hydrophila* challenge | [14] |
| *B. subtilis* WB60 | Japanese eel | 5×10^6^; 1×10^7^ | ↑ Lysozyme and MPO activities (serum); ↑ IgM expression (intestine) | ↑ Survival (+182%) at day 10 post *Vibrio anguilarum* challenge | [16] |
| *B. subtilis* WB60 | Japanese eel | 1×10^7^; 1×10^8^ | ↑ Lysozyme and MPO activities (serum); ↑ IgM expression (intestine) | ↑ Survival (+35% and +45%) at day 10 post *V. anguilarum* challenge | [15] |
| *B. subtilis* | Nile tilapia | 2.38×10^5^; 3.57×10^5^; 4.76×10^5^ | ↑ Lysozyme, alternative complement, total Ig, AKP, and respiratory burst activities (serum); ↑ Lysozyme activity, total Ig and alternative complement activities (skin mucus) | N/A | [21] |
| *B. subtilis* GPSAK9 | Hybrid grouper | 10^9^ | ↑ Lysozyme and IgM (serum, liver, intestine) levels, ↑ C3 and C4 (intestine) levels, ↑ IL-1β, IL-8, TNF-α, IL-10, TGF-β expression (intestine) | ↑ Survival (+150%) at day 7 post *Vibrio harveyi* challenge | [26] |
| *B. subtilis* | Olive flounder | 5×10^7^ | ↑ Lysozyme activity and Ig levels (serum) | ↑ Survival (+25%) at day 7 post *S. iniae* challenge (not significant) | [22] |
| *B. subtilis* E20 | Parrot fish | 10^7^ | ↑ Lysozyme, respiratory burst and phagocytic activities (serum) | ↓ Cumulative molarity (-52%) at day 7 post *Vibrio alginolyticus* challenge | [24] |
| *B. subtilis, B. subtilis* 6-3-1, *B. subtilis* HAINUP40 | Hybrid Grouper | 10^8^ | ↑ IL-10 expression (liver) | N/A | [7] |
| *B. subtilis* HAINUP40 | Nile tilapia | 10^8^ | ↑ Respiratory burst activity (leukocytes); ↑ lysozyme activity (serum) | ↓ Cumulative molarity (-53%) at 14 days post *Streptococcus agalactiae* challenge (significance not reported) | [8] |
| *B. subtilis* C-3102 | Red sea bream | 4.88×10^9^ | ↑ Lysozyme activity (serum, skin mucus); ↑ peroxidase, antiprotease, and bactericidal activities (serum) | N/A | [20] |
| *B. subtilis* C-3102 | Hybrid surubim | 8.4×10^6^; 1.7×10^7^; 3.3×10^7^;  3.5×10^7^ | ↑ Phagocytic activity | ↑ Survival post *A. hydrophila* challenge | [17] |
| *B. subtilis* C-3102 | Red sea bream | 10^5^ | ↑ Lysozyme activity (serum, mucus); ↑ bactericidal, peroxidase, and antiprotease activities (serum) | N/A | [19] |
| *B. subtilis* | Olive flounder | 10^8^ | ↑ Lysozyme, IgM and C3 levels (serum) | N/A | [12] |
| *B. licheniformis* | Nile tilapia | 8×10^6^ | ↑ Lysozyme activity and C3 content (serum) | ↑ Survival (+108%) at day 5 post *S. iniae* challenge | [39] |
| *B. licheniformis* | Rohu | 10^7^ | ↑ Lysozyme activity (serum, mucus), ↑ACP and antiprotease activities (serum), ↑ phagocytic activity and respiratory burst (head kidney leucocytes) | ↑ Survival (+91%) at day 10 post *A. hydrophila* challenge. ↑ Lysozyme activity (serum, mucus), ↑ ACP and antiprotease activities (serum), ↑ phagocytic activity and respiratory burst (head kidney leucocytes) | [34] |
| *B. licheniformis* KU556167 | Rohu | 10^7^ | ↑ Lysozyme, ACP, phagocytic and respiratory burst activities and ↑ IgM levels (serum) | ↑ Survival (+172%) at day 14 post *A. hydrophila* challenge | [33] |
| *B. licheniformis* SB3086 | Nile tilapia | 10^5^; 10^7^; 10^9^ | ↑ Ig levels, lysozyme, respiratory burst and phagocytic activities (serum) | ↓ Cumulative molarity (-16%, -26%, -44%) at 15 days post *S. aureus* challenge (significance not reported) | [35] |
| *B. licheniformis* ATCC 11946 | Northern whiting | 10^9^ | ↑ ACP and AKP activities (liver) | ↓ Cumulative molarity (-29.5%) at 14 days post *Vibrio harveyi* challenge | [40] |
| *B. licheniformis* Dahb1 | Nile tilapia | 10^5^; 10^7^ | ↑ Lysozyme, AKP and MPO activities (serum and skin mucus) | ↓ Cumulative molarity (-47.7% and 59.3%) at 10 days post *A. hydrophila* challenge (significance not reported) | [38] |
| *B. amyloliquefaciens* GB-9 | Hybrid sturgeon | 10^7^ | ↑ Lysozyme activity (serum and mucus); ↑ leukocyte phagocytosis; ↑ ACP activity (serum) | N/A | [48] |
| *B. amyloliquefaciens*  KU556165 | Rohu | 10^7^ | ↑ Lysozyme, ACP, phagocytic and respiratory burst activities and ↑ IgM levels (serum) | ↑ Survival (+140%) at day 14 post *A. hydrophila* challenge | [33] |
| *B. amyloliquefaciens* | Nile tilapia | 10^7^ | ↓ IL-6 and ↑ C3 gene expression (spleen) | N/A | [52] |
| *B. amyloliquefaciens* AV5 | Nile tilapia | 10^6^; 10^8^ | ↑ Lysozyme, MPO and AKP activities (mucus and serum) | N/A | [43] |
| *B. amyloliquefaciens* LSG2-8 | *Rhynchocypris lagowskii* | 10^6^; 10^7^; 10^8^; 10^9^ | N/A | ↓ Cumulative molarity (-14%, -42.6%, -48.3% and 28.9%) at 14 days post *A. hydrophila* challenge; ↑ lysozyme, C3, C4, and IgM activities (serum) | [44] |
| *B. amyloliquefaciens* | Yellow catfish | 10^6^ | ↑ Respiratory burst, lysozyme (plasma), IgM expression (intestine) | N/A | [46] |
| *B. amyloliquefaciens* CECT 5940 | Nile tilapia | 1×10^5^; 2×10^5^ | ↑ lysozyme activity (serum); ↑ innate immune cell counts (WBCs, monocytes lymphocytes) | N/A | [53, 55] |
| *B. amyloliquefaciens* CECT 5940 | Nile tilapia | 10^5^ | ↑ IgM (blood); ↑ TNF-α and ↓ HSP70 expression (liver) | N/A | [53] |
| *B. amyloliquefaciens* JFP2 | Rock bream | 1.4×10^6^ | ↑Lysozyme and respiratory burst activities (serum) | ↓ Cumulative molarity (-61.1%) at 20 days post *S. iniae* challenge | [64] |
| *B. velezensis* GPSAK4 | Hybrid grouper | 10^9^ | ↑ Lysozyme activity (serum, liver, intestine), ↑ IgM (serum, liver), C3, C4 (intestine), ↑ IL-1β, TNF-α, IL-10, TGF-β expression (intestine) | ↑ Survival (+73%) at day 7 post *Vibrio harveyi* challenge | [26] |
| *B. pumilus* 1486^T^ | Nile tilapia | 1.85×10^2^ | ↑ IgM, lysozyme and phagocytic activities (blood) | N/A | [61] |
| *B. coagulans* ATCC 7050 | Northern whiting | 10^9^ | ↑ ACP and AKP activities (liver, not significant) | ↓ Cumulative molarity (-26.5%) at 14 days post *V. harveyi* challenge | [40] |

**Note: ↑ or ↓ indicates significant (P < 0.05) increase or decrease compared with control unless specified. In the control group, fish were fed a basal diet without probiotics. ACP: Acid phosphatase; AKP: Alkaline phosphatase; C3: Complement component 3; C4: Complement component 4; IgM: Immunoglobulin M; IFN-γ: Interferon gamma; IL-1β: Interleukin-1 beta; IL-8: Interleukin-8; IL-10: Interleukin-10; MPO: Myeloperoxidase; RB: Respiratory burst; TGF-α: Transforming growth factor alpha; TGF-β: Transforming growth factor beta; TNF-α: Tumour necrosis factor alpha; WBCs: White blood cells.**

**References**

[1] Wang, J., D. Fan, R. Zhao, T. Lu, S. Li, and D. Wang. "Effects of Dietary Supplementation with Endogenous Probiotics Bacillus Subtilis on Growth Performance, Immune Response and Intestinal Histomorphology of Juvenile Rainbow Trout (Oncorhynchus Mykiss)." *Fishes* 9, no. 6 (2024).

[2] Wang, C., X. Hu, H. Tang, W. Ge, L. Di, J. Zou, Z. Cui, and A. Zhou. "Multiple Effects of Dietary Supplementation with Lactobacillus Reuteri and Bacillus Subtilis on the Growth, Immunity, and Metabolism of Largemouth Bass (Micropterus Salmoides)." *Developmental and Comparative Immunology* 160 (2024).

[3] Lin, Y. T., Y. C. Hung, L. H. Chen, K. T. Lee, and Y. S. Han. "Effects of Adding Bacillus Subtilis Natto Ntu-18 in Paste Feed on Growth, Intestinal Morphology, Gastrointestinal Microbiota Diversity, Immunity, and Disease Resistance of Anguilla Japonica Glass Eels." *Fish and Shellfish Immunology* 149 (2024).

[4] Liaqat, R., S. Fatima, W. Komal, Q. Minahal, Z. Kanwal, M. Suleman, and C. G. Carter. "Effects of *Bacillus Subtilis* as a Single Strain Probiotic on Growth, Disease Resistance and Immune Response of Striped Catfish (*Pangasius Hypophthalmus*)." *PLoS ONE* 19, no. 1 (2024).

[5] Hou, T., Z. Tang, Z. Wang, and C. Li. "Evaluation of the Potential Probiotic *Bacillus Subtilis* Isolated from Darkbarbel Catfish (*Pelteobagrus Fulvidraco*) on Growth Performance, Serum Immunity, and Disease Resistance of *Aeromonas Hydrophila*." *Journal of Fish Biology* 106, no. 2 (2024).

[6] Han, C., S. Song, C. Cui, Y. Cai, Y. Zhou, J. Wang, W. Bei*, et al.* "Strain-Specific Benefits of Bacillus Probiotics in Hybrid Grouper: Growth Enhancement, Metabolic Health, Immune Modulation, and Vibrio Harveyi Resistance." *Animals* 14, no. 7 (2024).

[7] Han, C., H. Shi, C. Cui, J. Wang, L. Li, W. Bei, Y. Cai, and S. Wang. "Strain-Specific Benefits of Bacillus on Growth, Intestinal Health, Immune Modulation, and Ammonia-Nitrogen Stress Resilience in Hybrid Grouper." *Antioxidants* 13, no. 3 (2024).

[8] Liu, H., S. Wang, Y. Cai, X. Guo, Z. Cao, Y. Zhang, S. Liu*, et al.* "Dietary Administration of Bacillus Subtilis Hainup40 Enhances Growth, Digestive Enzyme Activities, Innate Immune Responses and Disease Resistance of Tilapia, Oreochromis Niloticus." *Fish and Shellfish Immunology* 60 (2017): 326-333.

[9] Eissa, E. S. H., M. N. Monier, Y. M. Abd El-Aziz, S. Saadony, M. S. Abu Husein, O. H. Abd El Megeed, M. O. Alamoudi*, et al.* "The Efficacy of Dietary Commercial Probiotic (*Bacillus Subtilis*) on Growth Performance, Hemato-Biochemical Response, and Histological Status of Red Tilapia (*Oreochromis Sp.*)." *Journal of Applied Aquaculture* 37, no. 1 (2024): 45-46.

[10] Magouz, F., H. Abu-Ghanima, A. I. Zaineldin, M. S. Gewaily, A. Soliman, A. A. Amer, E. M. Moustafa*, et al.* "Dietary Bacillus Subtilis Relieved the Growth Retardation, Hepatic Failure, and Antioxidative Depression Induced by Ochratoxin a in Thinlip Mullet (Liza Ramada)." *Aquaculture Reports* 22 (2022).

[11] Wang, Y., Q. Wang, K. Xing, P. Jiang, and J. Wang. "Dietary Cinnamaldehyde and Bacillus Subtilis Improve Growth Performance, Digestive Enzyme Activity, and Antioxidant Capability and Shape Intestinal Microbiota in Tongue Sole, Cynoglossus Semilaevis." *Aquaculture* 531 (2021).

[12] Li, Y., Y. Yang, L. Song, J. Wang, Y. Hu, Q. Yang, P. Cheng, and J. Li. "Effects of Dietary Supplementation of Lactobacillus Plantarum and Bacillus Subtilis on Growth Performance, Survival, Immune Response, Antioxidant Capacity and Digestive Enzyme Activity in Olive Flounder (Paralichthys Olivaceus)." *Aquaculture and Fisheries* 6, no. 3 (2021): 283-288.

[13] Xue, J., K. Shen, Y. Hu, Y. Hu, V. Kumar, G. Yang, and C. Wen. "Effects of Dietary Bacillus Cereus, B. Subtilis, Paracoccus Marcusii, and Lactobacillus Plantarum Supplementation on the Growth, Immune Response, Antioxidant Capacity, and Intestinal Health of Juvenile Grass Carp (Ctenopharyngodon Idellus)." *Aquaculture Reports* 17 (2020).

[14] Won, S., A. Hamidoghli, W. Choi, Y. Park, W. J. Jang, I. S. Kong, and S. C. Bai. "Effects of Bacillus Subtilis Wb60 and Lactococcus Lactis on Growth, Immune Responses, Histology and Gene Expression in Nile Tilapia, Oreochromis Niloticus." *Microorganisms* 8, no. 1 (2020).

[15] Lee, S., K. Katya, Y. Park, S. Won, M. Seong, A. hamidoghli, and S. C. Bai. "Comparative Evaluation of Dietary Probiotics Bacillus Subtilis Wb60 and Lactobacillus Plantarum Kctc3928 on the Growth Performance, Immunological Parameters, Gut Morphology and Disease Resistance in Japanese Eel, Anguilla Japonica." *Fish and Shellfish Immunology* 61 (2017): 201-210.

[16] Lee, S., K. Katya, A. Hamidoghli, J. Hong, D. J. Kim, and S. C. Bai. "Synergistic Effects of Dietary Supplementation of Bacillus Subtilis Wb60 and Mannanoligosaccharide (Mos) on Growth Performance, Immunity and Disease Resistance in Japanese Eel, Anguilla Japonica." *Fish and Shellfish Immunology* 83 (2018): 283-291.

[17] Nunes, A. L., M. S. Owatari, R. A. Rodrigues, L. E. Fantini, R. Y. D. Kasai, M. L. Martins, J. L. P. Mouriño, and C. M. de Campos. "Effects of Bacillus Subtilis C-3102-Supplemented Diet on Growth, Non-Specific Immunity, Intestinal Morphometry and Resistance of Hybrid Juvenile Pseudoplatystoma Sp. Challenged with Aeromonas Hydrophila." *Aquaculture International* 28, no. 6 (2020): 2345-2361.

[18] Oliveira, F. C., M. P. Soares, B. P. N. Oliveira, F. Pilarski, and C. M. de Campos. "Dietary Administration of Bacillus Subtilis, Inulin and Its Synbiotic Combination Improves Growth and Mitigates Stress in Experimentally Infected Pseudoplatystoma Reticulatum." *Aquaculture Research* 53, no. 12 (2022): 4256-4265.

[19] Zaineldin, A. I., S. Hegazi, S. Koshio, M. Ishikawa, A. Bakr, A. M. S. El-Keredy, M. A. O. Dawood*, et al.* "Bacillus Subtilis as Probiotic Candidate for Red Sea Bream: Growth Performance, Oxidative Status, and Immune Response Traits." *Fish and Shellfish Immunology* 79 (2018): 303-312.

[20] Zaineldin, A. I., S. Hegazi, S. Koshio, M. Ishikawa, M. A. O. Dawood, S. Dossou, Z. Yukun, and K. Mzengereza. "Singular Effects of Bacillus Subtilis C-3102 or Saccharomyces Cerevisiae Type 1 on the Growth, Gut Morphology, Immunity, and Stress Resistance of Red Sea Bream (Pagrus Major)." *Annals of Animal Science* 21, no. 2 (2021): 589-608.

[21] Mohammadi, G., T. J. Adorian, and G. Rafiee. "Beneficial Effects of Bacillus Subtilis on Water Quality, Growth, Immune Responses, Endotoxemia and Protection against Lipopolysaccharide-Induced Damages in Oreochromis Niloticus under Biofloc Technology System." *Aquaculture Nutrition* 26, no. 5 (2020): 1476-1492.

[22] Lee, C., J. H. Cha, M. G. Kim, J. Shin, S. H. Woo, S. H. Kim, J. W. Kim, S. C. Ji, and K. J. Lee. "The Effects of Dietary Bacillus Subtilis on Immune Response, Hematological Parameters, Growth Performance, and Resistance of Juvenile Olive Flounder (Paralichthys Olivaceus) against Streptococcus Iniae." *Journal of the World Aquaculture Society* 51, no. 2 (2020): 551-562.

[23] Cao, H., R. Yu, Y. Zhang, B. Hu, S. Jian, C. Wen, K. Kajbaf, V. Kumar, and G. Yang. "Effects of Dietary Supplementation with Β-Glucan and Bacillus Subtilis on Growth, Fillet Quality, Immune Capacity, and Antioxidant Status of Pengze Crucian Carp (Carassius Auratus Var. Pengze)." *Aquaculture* 508 (2019): 106-112.

[24] Liu, C. H., K. Wu, T. W. Chu, and T. M. Wu. "Dietary Supplementation of Probiotic, Bacillus Subtilis E20, Enhances the Growth Performance and Disease Resistance against Vibrio Alginolyticus in Parrot Fish (Oplegnathus Fasciatus)." *Aquaculture International* 26, no. 1 (2018): 63-74.

[25] da Paixão, A. E. M., J. C. dos Santos, M. S. Pinto, D. S. P. Pereira, C. E. C. de Oliveira Ramos, R. B. Cerqueira, R. D. Navarro, and R. F. da Silva. "Effect of Commercial Probiotics (Bacillus Subtilis and Saccharomyces Cerevisiae) on Growth Performance, Body Composition, Hematology Parameters, and Disease Resistance against Streptococcus Agalactiae in Tambaqui (Colossoma Macropomum)." *Aquaculture International* 25, no. 6 (2017): 2035-2045.

[26] Amoah, K., B. Tan, S. Zhang, S. Chi, Q. Yang, H. Liu, Y. Yang, H. Zhang, and X. Dong. "Host Gut-Derived Bacillus Probiotics Supplementation Improves Growth Performance, Serum and Liver Immunity, Gut Health, and Resistive Capacity against Vibrio Harveyi Infection in Hybrid Grouper (♀Epinephelus Fuscoguttatus × ♂Epinephelus Lanceolatus)." *Animal Nutrition* 14 (2023): 163-184.

[27] Kuebutornye, F. K. A., J. Tang, J. Cai, H. Yu, Z. Wang, E. D. Abarike, Y. Lu, Y. Li, and G. Afriyie. "In Vivo Assessment of the Probiotic Potentials of Three Host-Associated Bacillus Species on Growth Performance, Health Status and Disease Resistance of Oreochromis Niloticus against Streptococcus Agalactiae." *Aquaculture* 527 (2020).

[28] Büyükdeveci, M. E., İ Cengizler, J. L. Balcázar, and İ Demirkale. "Effects of Two Host-Associated Probiotics Bacillus Mojavensis B191 and Bacillus Subtilis Mrs11 on Growth Performance, Intestinal Morphology, Expression of Immune-Related Genes and Disease Resistance of Nile Tilapia (Oreochromis Niloticus) against Streptococcus Iniae." *Developmental and Comparative Immunology* 138 (2023).

[29] Liao, Z., Y. Liu, H. Wei, X. He, Z. Wang, Z. Zhuang, W. Zhao*, et al.* "Effects of Dietary Supplementation of Bacillus Subtilis Dsm 32315 on Growth, Immune Response and Acute Ammonia Stress Tolerance of Nile Tilapia (Oreochromis Niloticus) Fed with High or Low Protein Diets." *Animal Nutrition* 15 (2023): 375-385.

[30] Du, R. Y., H. Q. Zhang, J. X. Chen, J. Zhu, J. Y. He, L. Luo, S. M. Lin, and Y. J. Chen. "Effects of Dietary Bacillus Subtilis Dsm 32315 Supplementation on the Growth, Immunity and Intestinal Morphology, Microbiota and Inflammatory Response of Juvenile Largemouth Bass Micropterus Salmoides." *Aquaculture Nutrition* 27, no. 6 (2021): 2119-2131.

[31] Addo, S., A. A. Carrias, M. A. Williams, M. R. Liles, J. S. Terhune, and D. A. Davis. "Effects of Bacillus Subtilis Strains on Growth, Immune Parameters, and Streptococcus Iniae Susceptibility in Nile Tilapia, Oreochromis Niloticus." *Journal of the World Aquaculture Society* 48, no. 2 (2017): 257-267.

[32] ———. "Effects of Bacillus Subtilis Strains and the Prebiotic Previda® on Growth, Immune Parameters and Susceptibility to Aeromonas Hydrophila Infection in Nile Tilapia, Oreochromis Niloticus." *Aquaculture Research* 48, no. 9 (2017): 4798-4810.

[33] Mukherjee, A., G. Chandra, and K. Ghosh. "Single or Conjoint Application of Autochthonous Bacillus Strains as Potential Probiotics: Effects on Growth, Feed Utilization, Immunity and Disease Resistance in Rohu, Labeo Rohita (Hamilton)." *Aquaculture* 512 (2019).

[34] Sukul, T., Z. A. Kari, G. Téllez-Isaías, and K. Ghosh. "Autochthonous Bacilli and Fructooligosaccharide as Functional Feed Additives Improve Growth, Feed Utilisation, Haemato-Immunological Parameters and Disease Resistance in Rohu, Labeo Rohita (Hamilton)." *Animals* 13, no. 16 (2023).

[35] Yaqub, A., M. N. Awan, M. Kamran, and I. Majeed. "Evaluation of Potential Applications of Dietary Probiotic (Bacillus Licheniformis Sb3086): Effect on Growth, Digestive Enzyme Activity, Hematological, Biochemical, and Immune Response of Tilapia (Oreochromis Mossambicus)." *Turkish Journal of Fisheries and Aquatic Sciences* 22, no. 5 (2022).

[36] Qin, L., J. Xiang, F. Xiong, G. Wang, H. Zou, W. Li, M. Li, and S. Wu. "Effects of Bacillus Licheniformis on the Growth, Antioxidant Capacity, Intestinal Barrier and Disease Resistance of Grass Carp (Ctenopharyngodon Idella)." *Fish and Shellfish Immunology* 97 (2020): 344-350.

[37] Midhun, S. J., S. Neethu, D. Arun, A. Vysakh, L. Divya, E. K. Radhakrishnan, and M. Jyothis. "Dietary Supplementation of Bacillus Licheniformis Hga8b Improves Growth Parameters, Enzymatic Profile and Gene Expression of Oreochromis Niloticus." *Aquaculture* 505 (2019): 289-296.

[38] Gobi, N., B. Vaseeharan, J. C. Chen, R. Rekha, S. Vijayakumar, M. Anjugam, and A. Iswarya. "Dietary Supplementation of Probiotic Bacillus Licheniformis Dahb1 Improves Growth Performance, Mucus and Serum Immune Parameters, Antioxidant Enzyme Activity as Well as Resistance against Aeromonas Hydrophila in Tilapia Oreochromis Mossambicus." *Fish and Shellfish Immunology* 74 (2018): 501-508.

[39] Han, B., W. Q. Long, J. Y. He, Y. J. Liu, Y. Q. Si, and L. X. Tian. "Effects of Dietary Bacillus Licheniformis on Growth Performance, Immunological Parameters, Intestinal Morphology and Resistance of Juvenile Nile Tilapia (Oreochromis Niloticus) to Challenge Infections." *Fish and Shellfish Immunology* 46, no. 2 (2015): 225-231.

[40] Amoah, K., X. H. Dong, B. P. Tan, S. Zhang, S. Y. Chi, Q. H. Yang, H. Y. Liu, Y. Z. Yang, and H. Zhang. "Effects of Three Probiotic Strains (Bacillus Coagulans, B. Licheniformis and Paenibacillus Polymyxa) on Growth, Immune Response, Gut Morphology and Microbiota, and Resistance against Vibrio Harveyi of Northern Whitings, Sillago Sihama Forsskál (1775)." *Animal Feed Science and Technology* 277 (2021).

[41] Taherpour, M., L. Roomiani, H. R. Islami, and M. S. Mehrgan. "Effect of Dietary Butyric Acid, Bacillus Licheniformis (Probiotic), and Their Combination on Hemato-Biochemical Indices, Antioxidant Enzymes, Immunological Parameters, and Growth Performance of Rainbow Trout (Oncorhynchus Mykiss)." *Aquaculture Reports* 30 (2023).

[42] Yousefi, M., M. Ahmadifar, S. Mohammadzadeh, N. Kalhor, D. E. Esfahani, A. Bagheri, N. Mashhadizadeh, M. S. Moghadam, and E. Ahmadifar. "Individual and Combined Effects of the Dietary Spirulina Platensis and Bacillus Licheniformis Supplementation on Growth Performance, Antioxidant Capacity, Innate Immunity, Relative Gene Expression and Resistance of Goldfish, Carassius Auratus to Aeromonas Hydrophila." *Fish and Shellfish Immunology* 127 (2022): 1070-1078.

[43] Shija, V. M., G. E. Zakaria, K. Amoah, L. Yi, J. Huang, F. Masanja, Z. Yong, and J. Cai. "Dietary Effects of Probiotic Bacteria, Bacillus Amyloliquefaciens Av5 on Growth, Serum and Mucus Immune Response, Metabolomics, and Lipid Metabolism in Nile Tilapia (Oreochromis Niloticus)." *Aquaculture Nutrition* 2024 (2024).

[44] Yu, M., Y. Zhang, D. Zhang, Q. Wang, G. Wang, M. Elsadek, Q. Yao, Y. Chen, and Z. Guo. "The Effect of Adding Bacillus Amyloliquefaciens Lsg2-8 in Diets on the Growth, Immune Function, Antioxidant Capacity, and Disease Resistance of Rhynchocypris Lagowskii." *Fish and Shellfish Immunology* 125 (2022): 258-265.

[45] Yu, M. N., Z. C. Wu, Y. R. Zhang, D. M. Zhang, Q. J. Wang, L. L. Lin, G. Q. Wang*, et al.* "Evaluation of Probiotic Potential and Fermentation Characteristics of Bacillus Amyloliquefaciens Lsg2-8 from Intestine of Rhynchocypris Lagowskii." *Aquaculture Research* 53, no. 5 (2022): 1873-1889.

[46] Xue, M., Y. Wu, Y. Hong, Y. Meng, C. Xu, N. Jiang, Y. Li*, et al.* "Effects of Dietary Bacillus Amyloliquefaciens on the Growth, Immune Responses, Intestinal Microbiota Composition and Disease Resistance of Yellow Catfish, Pelteobagrus Fulvidraco." *Frontiers in Cellular and Infection Microbiology* 12 (2022).

[47] Wu, J., G. Xu, Y. Jin, C. Sun, L. Zhou, G. Lin, R. Xu*, et al.* "Isolation and Characterization of Bacillus Sp. Gfp-2, a Novel Bacillus Strain with Antimicrobial Activities, from Whitespotted Bamboo Shark Intestine." *AMB Express* 8, no. 1 (2018): 84.

[48] Fei, H., G. D. Lin, C. C. Zheng, M. M. Huang, S. C. Qian, Z. J. Wu, C. Sun*, et al.* "Effects of Bacillus Amyloliquefaciens and Yarrowia Lipolytica Lipase 2 on Immunology and Growth Performance of Hybrid Sturgeon." *Fish and Shellfish Immunology* 82 (2018): 250-257.

[49] Kim, D. H., D. Subramanian, and M. S. Heo. "Dietary Effect of Probiotic Bacteria, Bacillus Amyloliquefaciens-Jfp2 on Growth and Innate Immune Response in Rock Bream *Oplegnathus Fasciatus*, Challenged with *Streptococcus Iniae*." *Israeli Journal of Aquaculture - Bamidgeh* 69, no. 1 (2017).

[50] Chistyakov, V., V. Melnikov, M. L. Chikindas, M. Khutsishvili, A. Chagelishvili, A. Bren, N. Kostina, V. Cavera, and V. Elisashvili. "Poultry-Beneficial Solid-State Bacillus Amyloliquefaciens B-1895 Fermented Soybean Formulation." *Biosci Microbiota Food Health* 34, no. 1 (2015): 25-28.

[51] Ponomareva, E. N., M. N. Sorokina, V. A. Grigoriev, M. Mazanko, V. A. Chistyakov, and D. V. Rudoy. "Probiotic Bacillus Amyloliquefaciens B-1895 Improved Growth of Juvenile Trout." *Food Science of Animal Resources* 44, no. 4 (2024): 805-816.

[52] Guan, M., J. Guan, H. Zhang, D. Peng, X. Wen, X. Zhang, and Q. Pan. "Effect of Moringa Oleifera, Bacillus Amyloliquefaciens, and Their Combination on Growth Performance, Digestive Enzymes, Immunity, and Microbiota in Nile Tilapia (Oreochromis Niloticus)." *Aquaculture Nutrition* 2024 (2024).

[53] Al-Deriny, S. H., M. A. O. Dawood, A. A. A. Zaid, W. F. El-Tras, B. A. Paray, H. Van Doan, and R. A. Mohamed. "The Synergistic Effects of Spirulina Platensis and Bacillus Amyloliquefaciens on the Growth Performance, Intestinal Histomorphology, and Immune Response of Nile Tilapia (Oreochromis Niloticus)." *Aquaculture Reports* 17 (2020).

[54] Authority, European Food Safety. "Safety and Efficacy of Ecobiol® (Bacillus Amyloliquefaciens) as Feed Additive for Chickens for Fattening - Scientific Opinion of the Panel on Additives and Products or Substances Used in Animal Feed." *EFSA Journal* 6, no. 8 (2008): 773.

[55] Ghalwash, H. R., A. S. Salah, A. M. El-Nokrashy, A. M. Abozeid, V. H. Zaki, and R. A. Mohamed. "Dietary Supplementation with Bacillus Species Improves Growth, Intestinal Histomorphology, Innate Immunity, Antioxidative Status and Expression of Growth and Appetite-Regulating Genes of Nile Tilapia Fingerlings." *Aquaculture Research* 53, no. 4 (2022): 1378-1394.

[56] Simó-Mirabet, P., M. C. Piazzon, J. A. Calduch-Giner, A. Ortiz, M. Puyalto, A. Sitjà-Bobadilla, and J. Pérez-Sánchez. "Sodium Salt Medium-Chain Fatty Acids and Bacillus-Based Probiotic Strategies to Improve Growth and Intestinal Health of Gilthead Sea Bream (Sparus Aurata)." *PeerJ* 2017, no. 12 (2017).

[57] Silva, T. F., T. R. Petrillo, J. Yunis-Aguinaga, P. F. Marcusso, G. da Silva Claudiano, F. R. de Moraes, and J. R. de Engrácia Moraes. "Effects of the Probiotic Bacillus Amyloliquefaciens on Growth Performance, Hematology and Intestinal Morphometry in Cage-Reared Nile Tilapia." *Latin American Journal of Aquatic Research* 43, no. 5 (2015): 963-971.

[58] Zhang, D., Y. Gao, X. Ke, M. Yi, Z. Liu, X. Han, C. Shi, and M. Lu. "*Bacillus Velezensis* Lf01: In Vitro Antimicrobial Activity against Fish Pathogens, Growth Performance Enhancement, and Disease Resistance against Streptococcosis in Nile Tilapia (*Oreochromis Niloticus*)." *Applied Microbiology and Biotechnology* 103 (2019): 9023-9035.

[59] Chang, X., M. Kang, L. Yun, Y. Shen, J. Feng, G. Yang, J. Zhang, and X. Meng. "Sodium Gluconate Increases Bacillus Velezensis R-71003 Growth to Improve the Health of the Intestinal Tract and Growth Performance in the Common Carp (Cyprinus Carpio L.)." *Aquaculture* 563 (2023).

[60] Munglue, P., K. Kronghinrach, K. Rattana, S. Sangchanjiradet, and K. Dasri. "Effect of Dietary Bacillus Pumilus A1_Ym_1 on Growth, Intestinal Morphology and Some Hematological Parameters of Hybrid Catfish (Clarias Macrocephalus × Clarias Gariepinus)." *Asia-Pacific Journal of Science and Technology* 24, no. 2 (2019).

[61] Hassaan, M. S., E. Y. Mohammady, M. R. Soaudy, M. A. Elashry, M. M. A. Moustafa, M. A. Wassel, H. A. S. El-Garhy, E. R. El-Haroun, and H. E. Elsaied. "Synergistic Effects of Bacillus Pumilus and Exogenous Protease on Nile Tilapia (Oreochromis Niloticus) Growth, Gut Microbes, Immune Response and Gene Expression Fed Plant Protein Diet." *Animal Feed Science and Technology* 275 (2021).

[62] Yu, Y., C. Wang, A. Wang, W. Yang, F. Lv, F. Liu, B. Liu, and C. Sun. "Effects of Various Feeding Patterns of Bacillus Coagulans on Growth Performance, Antioxidant Response and Nrf2-Keap1 Signaling Pathway in Juvenile Gibel Carp (Carassius Auratus Gibelio)." *Fish and Shellfish Immunology* 73 (2018): 75-83.

[63] Afrilasari, W., W. Widanarni, and A. Meryandini. "Effect of Probiotic Bacillus Megaterium Ptb 1.4 on the Population of Intestinal Microflora, Digestive Enzyme Activity and the Growth of Catfish (Clarias Sp.)." *HAYATI Journal of Biosciences* 23, no. 4 (2016): 168-172.

[64] Kim, D. H., D. Subramanian, and M. S. Heo. "Dietary Effect of Probiotic Bacteria, Bacillus Amyloliquefaciens-Jfp2 on Growth and Innate Immune Response in Rock Bream Oplegnathus Fasciatus, Challenged with Streptococcus Iniae." *Israeli Journal of Aquaculture - Bamidgeh* 69 (2017).
